# Opticool: Cutting-edge transgenic optical tools

**DOI:** 10.1371/journal.pgen.1011208

**Published:** 2024-03-22

**Authors:** Kelli D. Fenelon, Julia Krause, Theodora Koromila

**Affiliations:** 1 Department of Biology, University of Texas at Arlington, Arlington, Texas, United States of America; 2 School of Biology, Aristotle University of Thessaloniki, Thessaloniki, Greece; HudsonAlpha Institute for Biotechnology, UNITED STATES

## Abstract

Only a few short decades have passed since the sequencing of GFP, yet the modern repertoire of transgenically encoded optical tools implies an exponential proliferation of ever improving constructions to interrogate the subcellular environment. A myriad of tags for labeling proteins, RNA, or DNA have arisen in the last few decades, facilitating unprecedented visualization of subcellular components and processes. Development of a broad array of modern genetically encoded sensors allows real-time, in vivo detection of molecule levels, pH, forces, enzyme activity, and other subcellular and extracellular phenomena in ever expanding contexts. Optogenetic, genetically encoded optically controlled manipulation systems have gained traction in the biological research community and facilitate single-cell, real-time modulation of protein function in vivo in ever broadening, novel applications. While this field continues to explosively expand, references are needed to assist scientists seeking to use and improve these transgenic devices in new and exciting ways to interrogate development and disease. In this review, we endeavor to highlight the state and trajectory of the field of in vivo transgenic optical tools.

## Introduction

Biological inquiries utilizing live organisms offer superior relevancy to cell culture and fixed tissue studies in unraveling the expansive mysteries which remain in cell, developmental, and disease biology because the research is being performed in the most functionally relevant environment. A panoply of tools has been developed to interrogate these perplexities of nature, but none as exciting as the growing number of optical tools which have recently become available for use in live organism studies.

As such, this review will focus on those optical tools that can be readily used in transgenesis and will not cover the tools for interrogating biological processes via fixed samples, nonoptical means, or by using tools that require external biochemical treatments. In the first instance, there are several available extensive reviews covering advances in chromatin architectural characterization techniques [1,2], fluorescent in situ hybridization (FISH)-related methods [3,4], and transcriptomics [5,6] and proteomics [7,8]. Similarly, improvements in multiomics [9], mass spectrometry [10,11], DamID and BioID [12–14] have been recently chronicled. Exciting new in vivo dye, drug, and other external treatment techniques, while beyond the scope of this inquiry, are exploding in utility in basic and translational molecular biology fields.

Transgenic models recapitulate the natural settings of biological inquiries while reducing the frequency of required optimization steps and autonomously reproducing experimental toolkits. Here, we endeavor to compile a reference for anyone seeking to understand or develop new transgenic models expressing optical tools for visualization, quantification, and/or manipulation of subcellular and tissue-level molecular processes.

### 1 Advanced tools for visualizing subcellular functions in vivo

#### Fluorescent transgenic protein detection tags

**Fig 1:**
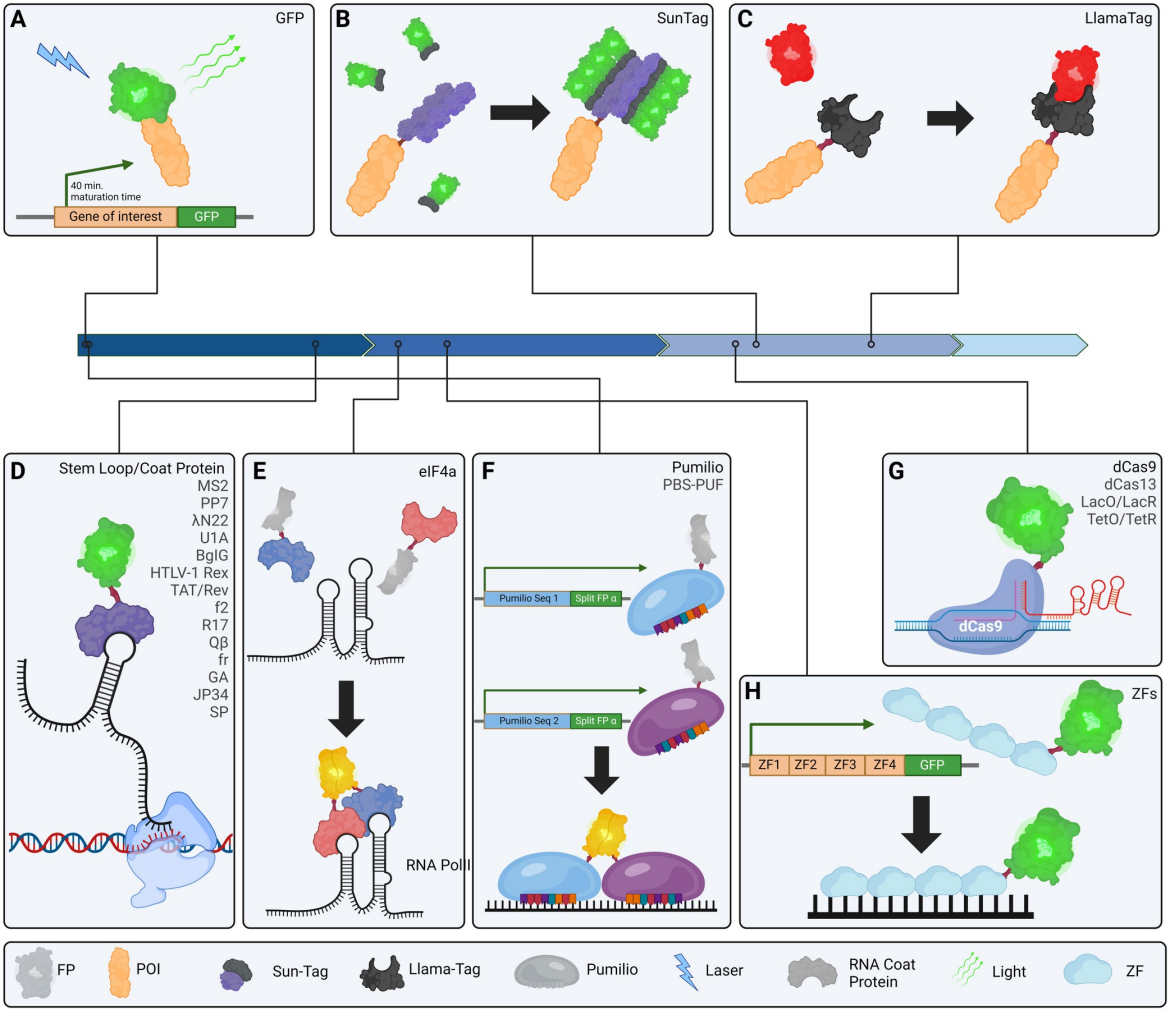
Tools for visualizing subcellular components in vivo. (A) Sequencing of GFP in 1992 allowed for creation of fluorescently labeled, transgenic labels and set in motion the progress of the field. (B) SunTag:FP fucion proteins bind to the SunTag scaffolding to brightly label POIs. (C) LlamaTag binds directly to specific FPs to rapidly label POIs. (D) Stem loop binding proteins are used to label RNA sequences. (E) elF4a is a two-part coat protein dimer that binds its specific stem loop structure and allows for split FP background elimination. (F) Pumilio allows for labeling of RNA sequencing through a genetically encodable 8 bp-binding region which can be customized. It has been improved to work in pairs attached to split FPs to eliminate background fluorescence and increase sensitivity. (G) dCas9 can be used to label DNA sequences in vivo through gRNA targeting. (H) Zinc fingers can be genetically engineered to target DNA sequences in vivo and label them with FPs. Blue arrows represent time in decades since the sequencing of GFP in 1990, left, to the present, right. FP, fluorescent protein; POI, protein of interest.

While GFP has only been used as a transgenic tool for less than 30 years [15], transgenic fluorescent markers for molecular biology have advanced to the point that mere labeling of proteins with even some of the most optimized fluorophores [16,17] is sometimes regarded as banal with growing criticisms of these fluorescent proteins’ (FP) limitations (Fig 1A). Maturation rates of FPs have been a particularly popular target of recent optimization studies to ameliorate unavoidable delays in protein detection of target-FP fusions [18]. However, such limitations are now being avoided altogether through an alternative approach whereby FPs are ubiquitously expressed separately from the target protein but directed to the target protein through tag binding in vivo. In this way, fluorescence can be detected immediately upon translation of the target protein.

These secondary attachment fluorescent systems are generally termed tags [18–21], with one of the most popular transgenic tag systems being Sun-tag [19,21]. The Sun-tag system makes use of 2 short peptides with high affinity to one another to recruit FPs to target proteins. A fusion protein is constructed consisting of a POI and up to 24 repeating sequences that allow for complimentary attachment via a second FP fusion protein [22,23]. Specifically, a single-chain fragment variable (ScFv) antibody is used which recognizes GCN4; the GCN4 repeats fused to the POI are used to recruit ScFv-bound FPs to tag the POI for detection (Fig 1B). Sun-tag’s “scaffolding system” produces increased brightness because several fluorescent molecules can simultaneously attach to the POI [21]. Llama-tags are another protein tagging system allowing for faster visualization in vivo due to their use of already matured FPs [18]. Llama-tags use optimized nanobody fusion proteins to directly recruit FPs to POIs (Fig 1C). This mechanism therefore requires creation of only 1 transgene to be used and allows for transgenic addition of the tags into FP-expressing animals which are now ubiquitous [24,25].

#### Real-time transcriptional visualization

Rapid fluorescence detection via pooled mature fluorophores at the ready has grown in popularity not only for visualizing protein locations, but also for detecting gene expression and mRNA dynamics in vivo [19,26,27]. By far the most popular systems for real-time transcription detection are the MS2 [28,29] and PP7 [30–33] systems, whereby MS2 coat protein (MCP) and PP7 coat protein (PCP), fused to fluorophores, bind MS2 and PP7 RNA stem loops, respectively. These have been used to generate a broad array of transgenic animals and computational tools to track gene expression timing in live imaging experiments [19,34–39]. In addition to these popular mRNA visualization options, multiple other aptamer-coat protein pairs are currently available for transgenic studies including BglG [40], U1Ap [41], *λ*N22 [42], HTLV-1 Rex [43], TAT/REV [44], and several less tested variants [40,45,46] (Fig 1D).

The two-part aptamer-binding eIF4a [47] provides an intriguing twist on the system, providing a reliable, straight-forward method to label RNA without ubiquitous background fluorescence by fusing the 2 parts to split fluorophores [48,49] (Fig 1E). Further, RNA-binding Pumilio [50,51] allows for sequence modification to bind to chosen, engineered eight-nucleotide sequences of RNA and has been implemented in the Pumby [52] labeling system to label adjacent mRNA sites to utilize split fluorophores (Fig 1F). The major limitation of most tag systems being the necessity for high background fluorescent signal from a pool of overexpressed FPs poised for binding to the tag, these split fluorophore approaches present significant advancement by eliminating this excess fluorescent noise. Similarly, DNA sequences can be targeted transgenically for visualization using dCAS9-FP fusions to target sequences in a guide RNA directed manner [53] (Fig 1G) or via traditional engineering of zinc finger-FP fusions [54] (Fig 1H).

### 2 Transgenic optical sensors

#### FRET-based sensors

**Fig 2:**
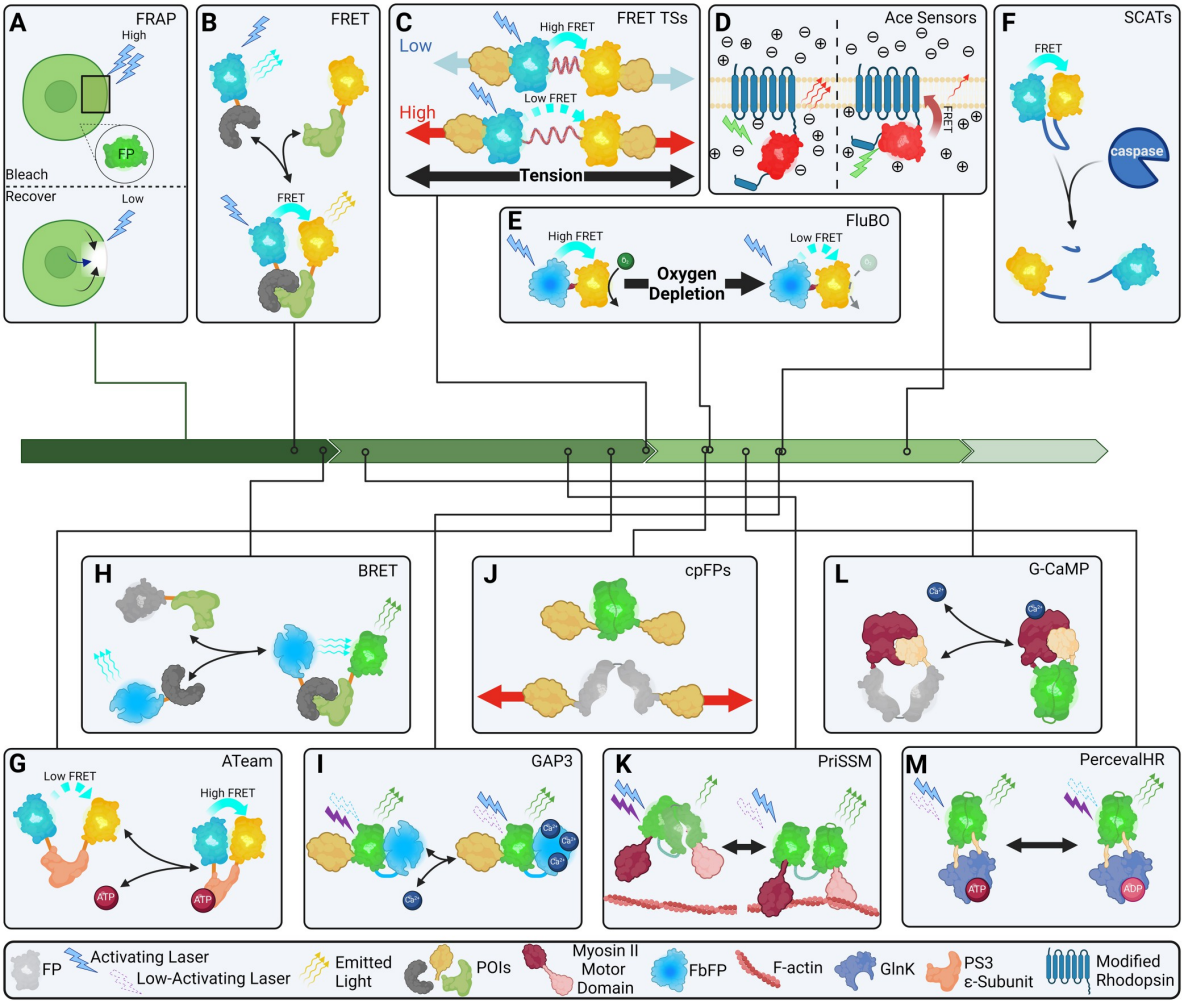
Transgenic optical sensors. (A) One of the earliest subcellular sensor system, FRAP is used to detect protein diffusion and translation rates in vivo through photobleaching of an FP, followed by imaging of fluorescent recovery. (B) Fluorophores with overlapping excitation and emission wavelengths can share energy through FRET when sufficiently close in proportion to their distance from one another. (C) Separating FRET pairs with molecular springs allows for measurement of extension of the molecular springs, and therefore, tension. (D) Ace Sensors utilize modified rhodopsin that alters configuration upon formation of membrane potential altering the FRET efficiency of an inserted FP. (E) Because flavin-binding FP fluorescence maturation is oxygen independent but GFP-based FPs are not, FluBO reports O_2_ levels via O_2_-dependent FRET efficiency variation. (F) Separating a FRET pair with a caspase recognition sequence allows for detection of caspase activity via SCAT sensors. (G) Steric alteration of the PS3 ε-subunit when bound to ATP allows the ATeam ATP sensor to detect ATP via FRET efficiency alteration. (H) Similar to FRET, luminescing proteins can sometimes share energy. (I) The GAP sensor utilizes shift of GFP excitation wavelength when in proximity to aequorin when bound or unbound to calcium ions. (J) The biochemical domains of some FPs can be isolated, allowing for artificially induced fragility. (K) Detection of F-actin polymerization with PriSSM is achieved by wedging a GFP and cpGFP into a Myosin II motor domain such that binding of the motor domain to F-actin produces a steric alteration which increases fluorescence and increases blue:near-IF excitation ratio. (L) In G-CaMP, a cpGFP is flanked by Calmodulin and a myosin light chain fragment which bind one another such that steric conditions disrupt fluorescence in the absence of calcium binding. (M) ADP/ATP steric alterations in GlnK fluctuate fused cpGFP optimal excitation wavelength of the Perceval sensor. Green arrows represent time in decades since the sequencing of GFP in 1990, left, to the present, right. FP, fluorescent protein; FRAP, fluorescence recovery after photobleaching; FRET, Förster resonance energy transfer.

Beyond simply labeling cellular components, a growing array of transgenic optical tools for measuring subcellular and tissue conditions have arisen in recent decades [55,56]. The field of transgenic biosensors has, again, bounded forward from the earliest methods, such as fluorescence recovery after photobleaching (FRAP, Fig 2A), in a mere few decades [57,58]. These remarkable nano- and micro-scale auto-biological devices present a unique opportunity in the fields of developmental and disease biology allowing for measurements where even the most modern and advanced exogenous technologies are insufficient [59].

Perhaps the most versatile of these transgenic instruments is proximity detection via Förster resonance energy transfer (FRET) [60–62]. In this method, 2 proteins are labeled with fluorophores capable of FRETing with one another and the distance between them can be calculated when they’re in sufficient proximity to one another [63] (Fig 2B). Mechanical forces are known to be significant in several developmental and disease pathways [64–71]. A particularly innovative extension of this method is the creation of several tension sensors [72–77] (Fig 2C). Because the distance between 2 FRETing fluorophores can be detected optically, fusing compatible fluorophores together separated by a molecular spring, generally spectrin [78] or flagelliform [79] repeats, allows for the measurement of forces on the FRET pairs via a simple calculation of the measured distance and the known spring-like characteristics of the separator [80–82]. These sensors have been developed in mechanical components of cells and tissues ranging from extracellular matrix and cell–cell connections [83,84], cytoskeletal connections [85,86], and all the way into the nucleus and onto the genome itself [75].

An innovative use of FRET in sensors has facilitated the detection of membrane voltage potentials via opsin-based Acetabularia opsin (Ace) sensors. In the Ace sensor systems, an FP is fused to an opsin with which it can FRET; when the intracellular side of the membrane gains a net positive charge, increased FRET reduces fluorescence of the FP (Fig 2D). Ace sensors now come in a variety of spectral flavors including red (VARNAM [87]) and green (Ace2N-mNeon [88]). Another creative use of FRET to sense subcellular conditions is FluBO [89]. While optical sensing tools have long been a field of inquiry and optimization [90], FluBO cleverly uses a flavin-binding FP (FbFP) as donor with a YFP acceptor to detect molecular oxygen (O_2_) within cells. YFP is sensitive to O_2_ levels while FbFP is not, allowing for detection of higher FRET efficiency in O_2_ rich cells (Fig 2E). Caspase activity can further be measured using FRET pairs separated by caspase cleavage sites thereby providing a reduction in FRET as a readout for caspase activity [91–97] (see Fig 2F). ATP concentration is an important subcellular condition which has motivated the creation of a multitude of sensors [98]. The transgenic ATeam sensors [99] utilize modified ATP synthase ε-subunit transversely terminally fused to a donor and acceptor FPs such that binding to ATP [99–101] or MgATP [102] brings the FPs into proximity whereby increased FRET efficiency can be detected (Fig 2G).

#### Bioluminescence-based sensors

A somewhat similar method for functional and proximity detection to the FRET-based sensors is the use of bioluminescent resonant energy transfer (BRET) whereby a bioluminescent molecule is used to shift the emission fluorescence of a fluorophore in sufficient proximity via molecular transference of the bioluminescent enzyme’s emission to the fluorophore [103,104] (Fig 2H). FRET detection in FRET sensors, while precise, has proven difficult to effectively implement in vivo as autofluorescence and relatively weak signal-to-noise making alternatives like BRET attractive [105,106].

However, while a panoply of BRET sensors exist [106,107], e.g., Ca^2+^ sensing with LuCID [108], cAMP sensing with CAMYEL [109,110], cytoskeletal tension sensing [111], O_2_ sensing [112], caspase activity sensing [113], and POI/POI interaction detection [114], current iterations remain shackled to the requirement of exogenous introduction of their cofactor substrates [115]. As such, these tools remain distinct from pure transgenic optical tools. Despite this, recent advances have been made to achieve autoluminescence from bioluminescence systems by introducing genes that ultimately catalyze endogenous synthesis of chemical cofactors [116–119]. Conversely, a clever employment of *Aequorea victorea* apo-aequorin allows for Ca^2+^ sensing without its bioluminescent cofactor requirement in the GAP (GFP and apo-aequorin) sensors [120]. In these sensors, binding of calcium ions to aequorin shifts the excitation maximum of GFP allowing for Ca^2+^ concentration quantification via ratiometric fluorescence measurement (Fig 2I).

#### Modified and permutated fluorophore sensors

A methodological extension of the split-fluorophore mechanism described above for in vivo tagging of RNA with low background is a class of sensors utilizing split-fluorophores [48,49]. In most of these systems, interactions of proteins labeled with complimentary subunits of the split fluorophores are detected by fluorescence via sufficient proximity of the subunits. While this method is similar to the FRET pair systems above, it has significant benefits and drawbacks in comparison: split-fluorophores confer a binary interaction measurement and not a distance, but offer higher detection resolution with less background noise and require less sophisticated imaging and analysis techniques than FRET systems. Thus, split-fluorophores have also been used to develop binary force sensors where the subunits are separated by a flexible linker allowing for subunit separation upon sufficient tension [76,121].

Fluorophores have further been modified by rearrangement and insertions in their amino acid sequences to produce various effects, termed circularly permutated fluorophores (cpFPs) [122,123] (Fig 2J). A remarkable application of one such cpFP is the strain sensor PriSSM [124]. Proximity imaging (PRIM) [125]-based strain sensor module (PriSSM) facilitates detection of F-actin-myosin II strain via ratiometric fluorescence following 490 or 390 nm laser activation. Strain on PriSSM changes the orientations of tandem, contacting GFP and cpGFP FPs making the optimal activation wavelength 390 nm and 490 nm detached from F-actin or under strain, respectively, as well as increasing fluorescence under strain (Fig 2K). A further implementation of a circularly permutated GFP (cpEGFP) was used to construct a Ca^2+^ sensor, G-Camp [17,126], which increases in fluorescence due to conformational changes induced by calcium ion binding (Fig 2L). The reduced efficiency of cpEGFP in the unbound state has the additional, key advantage of increasing the signal-to-noise ratio of G-CaMP relative to alternative strategies. The Perceval sensor [127–129] permits in vivo measurement of ATP/ADP ratios via measurement of fluorescence ratio from a circularly permuted monomeric Venus (cpmVenus) fused to *Methanococcus jannaschii* GlnK1 when excited by 490 or 405 nm wavelength lasers (Fig 2M). Further, use of modified FPs to be sensitive to other molecules has also been accomplished as in ClopHensor which uses a modified, chloride-ion-sensitive GFP, E^2^GFP, fused to DsRed-m to detect chloride levels in cells by ratiometric fluorescence [130,131].

### 3 Optogenetics: Optical tools for real-time subcellular manipulations

#### Activation/Inactivation systems

Optogenetics is a more recent technique that facilitates convenient manipulation of cellular function [132–136]. With the use of light, proteins can be utilized to affect the function of the cell. Optogenetics makes use of light-sensitive proteins (either artificial or naturally occurring) and adjusts their functionality through adjusting their secondary, tertiary, or quaternary structure. There is a myriad of mechanisms that can be affected with optogenetic tools, but all are initiated by a chromophore or light-absorbing amino acid. Optogenetics is commonly preferred over chemically inducible systems for a multitude of reasons. Specifically, optogenetic tools allow for greater specificity through avoidance of secondary chemical effects, availability of multiple isolated wavelengths allows for easy combinatorial implementations, modern laser microscopy enables precise 3D localization of effect, and physical barriers can often be overcome through the use of light rather than chemical compounds. These tools can often be adjusted within milliseconds allowing for specified cellular manipulation and can be used to study a broad range of cell types in live animals, especially during development when tissues depth is minimal and tissue transparency is greatest.

Inactivation of proteins by exposure to specific wavelengths of light is a straight-forward and reliable mechanism to modulate protein function in vivo. Degrons are naturally occurring peptide markers which facilitate degradation of the protein to which they are attached [137]. To be used as optogenetic tools, these degradation tags are fused to cryptochromes, enzymes which change conformation in response to light to expose or conceal cryptic domains within themselves, allowing for concealment and longevity of the target protein or exposure and degradation of the target protein in response to light.

**Fig 3:**
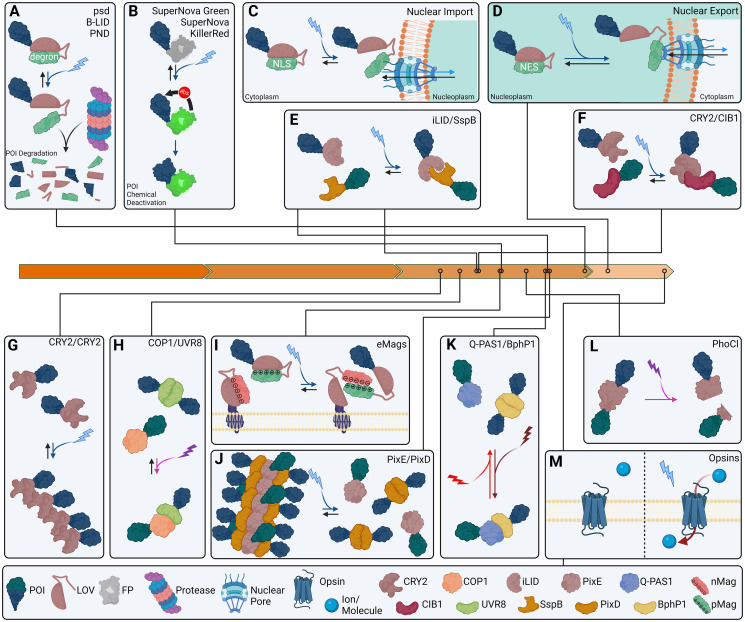
Optogenetic tools for real-time subcellular manipulations. (A) Degrons are used with LOV2 to yield blue light triggering of fusion protein degradation. (B) Photosensitizers are FPs which produce ROS biproducts when excited and can be used to distort fused proteins. (C) LANS and LINuS trigger reversible nuclear import upon blue light exposure by exposure of a cryptic NLS in a LOV2 fusion. (D) LINX and LEXY trigger reversible nuclear export upon blue light exposure by exposure of a cryptic NES in a LOV2 fusion. (E) iLID/SspB heterodimerize reversibly when exposed to blue light. (F) CRY2/CIB1 reversibly heterodimerize when exposed to blue light. It is important to remember that CRY2 also oligomerizes under blue light exposure. (G) Reversible CRY homooligomerization occurs when exposed to blue light. (H) COP1/UVR8 reversibly heterodimerizes when exposed to ultraviolet light. In the dark, UVR8 reversibly forms homodimers. (I) Positive (pMag) and negative (nMag) domains within Magnets tools dimerize in the presence of blue light via exposure from cryptic domains within LOV2 fusions. (J) PixE/PixD heterooligomerizes in the dark and can be reversibly dissociated into PixD homodimers and PixE monomers by exposure to blue light. (K) Q-PAS1/BphP1 heterodimers are reversibly formed when exposed to far red light, whereas BphP1 homodimers and Q-PAS1 monomers are produced via red light or darkness. (L) PhotoCleavable cleaves upon exposure to violet light. (M) Opsin membrane transport pumps are used to induce transmembrane pumping of ions when exposed to appropriate wavelengths of light. Orange arrows represent time in decades since the sequencing of GFP in 1990, left, to the present, right. FP, fluorescent protein; LANS, light-activated nuclear shuttle; LOV2, light oxygen voltage 2; ROS, reactive oxygen species.

While optogenetic degrons, as a class, are often referred to as photo-sensitive degrons, psd is a specific optogenetic degron developed by fusing the photosensitive domain of *Arabidopsis thaliana* phototropin1, light oxygen voltage 2 (LOV2), to mouse ornithine decarboxylase carboxy-terminal degron (cODC) [138,139]. When fused to a protein of interest (POI), psd exposure to blue light induces a conformational change to expose the cryptic degron, leading to protease degradation of the construct which can then be repopulated in the dark (Fig 3A). Another system developed for triggering protease degradation of fusion proteins by exposure to light is the blue light inducible degradation (B-LID) method [140]. B-LID contains a small peptide degron [141] rendered cryptic by fusion to a LOV2 domain; in this way, degradation of fusion proteins is triggered by exposure of the degron upon exposure to blue light (see Fig 3A). Photo-N-degron was recently developed to take advantage of light-dependent N-end rule-mediated protein degradation [142]. Light-induced uncoiling of the Jα helix in LOV2 exposes an N-terminal arginine amino acid and triggers N-end rule degradation [143,144] (see Fig 3A).

Apart from degrons, there are other strategies for deactivation of proteins. One such method is photosensitizers used in chromophore-assisted light inactivation (CALI) [145,146]. Photosensitizers are chromophores which produce reactive oxygen species (ROS) in response to light activation [147]. CALI takes advantage of this production to inactivate proteins attached to a photosensitizer. The first genetically encoded photosensitizer was KillerRed, derived from the hydrozoan chromoprotein anm2CP, which produces phototoxic affects via ROS production in response to green light exposure [148]. KillerRed is an effective optogenetic tool for selectively killing cells and tissues through green light-mediated ROS production but is also a useful tool in CALI schemes (Fig 3B). Because CALI is a more direct system than degrons, inactivation is substantially more rapid than degron-based systems but have the drawback of producing cytotoxic ROS. Since KillerRed, multiple alternate photosensitizers have been developed to counter KillerRed’s propensity to dimerize and facilitate use of other wavelengths of light [149,150]. LightsOut introduces an AsLOV2 domain into the Gal4 transcription factor (TF), widely used for conditional transgene expression [151,152], between DNA-binding and gene activation domains to suppress Gal4-mediated expression when exposed to blue light [153].

#### Subcellular translocation systems

AsLOV2 has further been implemented in light-activated nuclear shuttle (LANS) [154] and light-inducible nuclear localization signal (LINuS) [155] to enable conditional nuclear localization of POIs upon exposure to blue light using a cryptic NLS (see Fig 3C). Conversely, the light-inducible nuclear export systems LINX [156,157] and LEXY [158,159] employ a light-exposable cryptic NES within an AsLOV2 fusion allowing for blue light-induced nuclear export of POIs (Fig 3D).

The ability to reversibly trigger *Botrytis cinerea* BcLOV4 membrane binding via blue light exposure has enabled its use as a conditional fusion protein localization system whereby blue light is used to target fusion proteins to the membrane [160–163]. An interesting, naturally occurring optogenetic tool for localization to DNA is *Erythrobacter litoralis* EL222 which dimerizes and binds to DNA in response to blue light [164,165]. This system is currently used in multiple contexts to control transcription via light exposure [166–170].

#### Binding/Polymerization systems

An enormously useful optogenetic innovation has been transgenic tools which facilitate manipulation of binding dynamics of POIs [171]. It is now possible to use these tools to hold proteins in contact with one another, sequester them against membranes, or release them via exposure to laser light (Fig 3E–3K). This mechanism is not only of inherent utility in POI functional control but also has been shown extensively to be greatly advantageous as a compounding factor in conjunction with other optogenetic mechanisms [132].

iLID, an improved and modified variant of LID [141], is a blue light inducible dimerizing protein which forms a dimer with SspB in light and dissociates in the dark [172] (Fig 3E). iLID utilizes an AsLOV2 domain fused to an SsrA peptide which is then able to bind with its binding partner, SspB [173], upon exposure via reversible, light-induced LOV2 conformational transformation. A deservedly popular use of the iLID system is OptoSOS [174–178]. OptoSOS utilizes a membrane-anchored iLID to recruit an SspB-fused SOS to trigger a Ras/Erk cascade when exposed to blue light. Another light-triggered dimerization system is cryptochrome 2 (CRY2) and CIB1 [179]. In this system, blue light induces heterodimerization of *Arabidopsis thaliana* CRY2/CIB1 (Fig 3F). Simultaneously, however, this system also causes CRY2 homo-oligomerization creating both a potential experimental challenge and a useful mechanistic expansion beyond other hetero/homodimerization systems [180–182] (Fig 3G). Intriguingly, an optogenetic heterodimerization system exists which utilizes UV-B light [183]. In the presence of UV-B, *Arabidopsis* COP1 binds to UVR8 and can be used bring POIs into proximity to one another in living cells (Fig 3H).

Similarly, heterodimerization can be controlled by red and far-red light, as in the phytochrome B (PhyB)/phytochrome interacting factor (PIF) system [184]. However, these red or far-red phytochrome systems require plant chromophore 3-Z phycocyanobilin (PCB) which is not naturally synthesized in most animals, making them challenging to use in transgenic systems. Nevertheless, PCB can be synthesized in mammalian cells by transgenically introducing 4 genes required to generate it from heme [118,119]. An intriguing use of this system is SOScat which operates identically to the iLID optoSOS system via red and far-red light rather than blue, allowing for greater freedom in designing combinatorial transgenic systems [185]. Furthermore, *Arabidopsis thaliana* PhyA/FHY1 behave very similarly allowing for the creation of REDMAP which facilitates gene activation under red light through heterodimerization and deactivation under far-red light via dissociation [186]. Bacteriophytochrome BphP1 is used as a light-sensitive binding partner to *Rhodopseudomonas palustris* PpsR2 [187]. More recently, a truncated variant of PpsR2, Q-PAS1, was engineered to reduce its size and mitigate natural oligomerization of PpsR2 [188,189]. In this light-sensitive binding method, far-red light exposure causes binding of BphP1 to its binding partner which is reversed in red light or darkness conditions (Fig 3K).

A further innovation in light-dependent interaction, Magnets, has been developed to induce POIs heterodimerization in the presence of blue light [190,191]. Magnets was developed by modifying the Per-Arnt-Sim (PAS) domain of the *Neurospora crassa* LOV protein Vivid which forms heterodimers when exposed to blue light [192–194]. Modifications were made to the protein sequence in the binding domain to either introduce positive (pMag) or negative (nMag) amino acids, thereby creating 2 modified Vivid photoreceptors which heterodimerize but do not homodimerize in the presence of blue light (Fig 3I). Another tool derived from Vivid is LightOn which uses Gal4 fused to Vivid and a p65 transactivation domain, GAVP, to induce dimerization and activation of UAS target gene expression when exposed to blue light [195].

Contrary to the bind-in-the-light/dissociate-in-the-dark scheme of the systems discussed so far, there also exist systems which employ the contrary scheme. An example of this is the *Synechocystis* PixD/PixE system. In the dark, PixE and PixD form oligomers containing 5 PixE and 10 PixD proteins which dissociate into PixE monomers and PixD homodimers when exposed to blue light [196] (Fig 3J). Interesting, bacterial photoactivated adenylyl cyclase (bPAC) [197], which utilizes a BLUF (blue light receptor using FAD) light sensor domain similarly to PixD, facilitates light-dependent increase in cAMP levels. In LOV2 trap and release of protein (LOVTRAP), a small protein, Zdark (Zdk), was developed to bind to the dark state of LOV2 [198]. This facilitates the conditional tethering and release of LOV2 to Zdk under dark or blue light exposure, respectively. Somewhat orthogonally paralogous to the Zdk/LOV2 binding pair is the Erbin PDZ/LOV2 binding pair, TULIPS [199], whereby a cpePDZ [200] can bind to a short peptide sequence fused to LOV2 upon blue light exposure. A disparate mechanism of light-mediated anchoring control is through light-controlled protein cleavage. An example of this system is PhotoCleavable (PhoCl) [201] (Fig 3L). PhoCl consists of a modified circularly permutated mMaple which spontaneously dissociates upon exposure to violet light and disconnection of N- and C- terminal fused POIs.

#### Opsins

Perhaps the simplest examples of optogenetic tools are opsins which have gained immense popularity in neuron studies and are lurching towards translational implementation. Opsins are light-sensitive membrane proteins which regulate transmembrane ion transfer. This makes them simple, inherent optogenetic tools whereby membrane potential can be controlled by light exposure [202] (Fig 3M).

An example of an opsin optogenetic tool is pHoenex, an optical proton pump tool [203]. pHoenex uses a modified *Halorubrum sodomense* Archaerhodopsin-3 (Arch3) light-sensitive proton pump fused to an ATPase, vesicular protein Synaptophysin, and optical pH sensor pHluorin [204] to facilitate H^+^ shuttling to acidify the interior of synaptic vesicles via yellow light and pH detection via blue light. Similarly, several other optogenetic opsin tools are available which facilitate light-mediated transmembrane proton pumping [205], sodium ion pumping [206], and chloride ion pumping [207,208]. Furthermore, guanylyl and adenylyl cyclase rhodopsins, such as rhodopsin-guanylyl cyclase (RhGC) [209], rhodopsin cyclic nucleotide phosphodiesterase (Rh-PDE) [210], and guanylyl cyclase rhodopsin (CyclOp) [211], have demonstrated utility in mediating light-dependent cAMP and/or cGMP levels to exogenously regulate subcellular signaling.

### 4 Modern advances in combinatorial, real-time optical methods

Techniques involving simple, direct light-dependent messenger manipulation are quite popular and elegant; however, the most exciting implementations of transgenic optical tools are those which involve cooperative implementation of multiple systems simultaneously to enable greater control over and comprehension of the molecular biological systems being interrogated. It is feasible that experimental protocols can already be developed to visualize, measure, and manipulate one or more subcellular molecular processes in the same cell(s) in real- or near-real-time. Indeed, a growing number of such systems combining multiple transgenic optical tools and strategies are being developed to combine or improve upon individual schemes.

**Fig 4:**
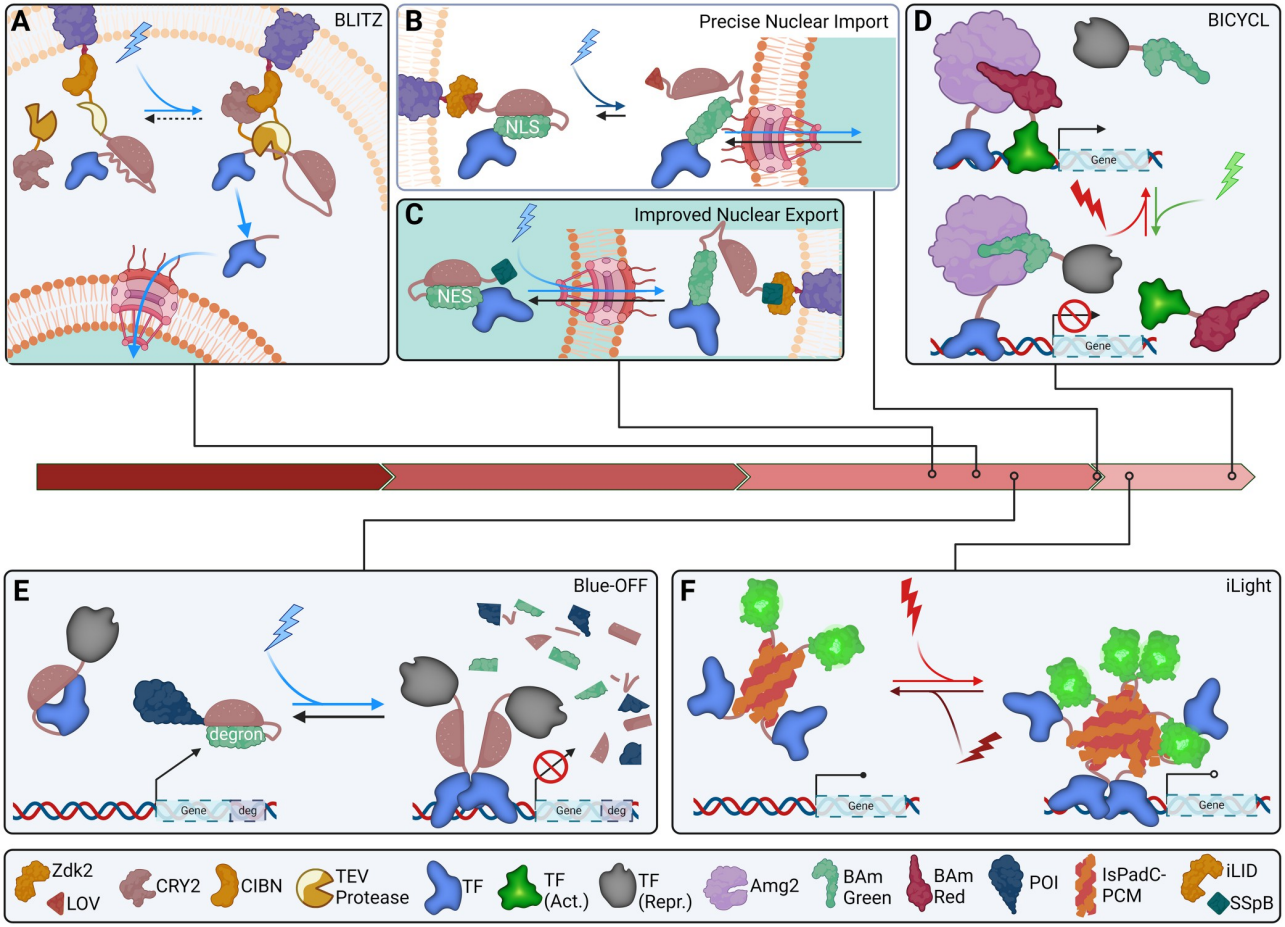
Modern advances in combinatorial, real-time optical methods. (A) The BLITZ system uses CRY2/CIBN blue light-induced dimerization to connect TEV fragments which are then able to free a membrane-anchored factor by cleaving a cryptic recognition site within a LOV2 domain. (B) LANSTRAP and CLASP improve upon LANS/LiNUS by introducing membrane sequestration of cytoplasmic fusion proteins via Zdk/LOV to more reliably and constantly maintain nuclear exclusion in the absence of blue light. (C) LINXnano improves upon the LINX/LEXY systems by fusing LINX to a minimal version of SspB, nano, which binds to membrane-tethered iLID in the presence of blue light. (D) Amg2 of the BICYCL system reversibly binds BAm Red when exposed to red light and BAm Green when exposed to green light, facilitating wavelength-directed transcriptional toggling by recruiting either activators or repressors to genomic regulatory regions. (E) The Blue-OFF system triggers transcriptional silencing and simultaneous degradation of a POI by combining an optogenetic degron fused to the POI with a repressor-bound LOV2 harboring a cryptic DNA-binding protein. (F) iLight utilizes IsPadC red-light-inducible homodimerization and isomeric steric hindrance of fused proteins in dark or far-red conditions to reversibly recruit TFs to gene loci. Red arrows represent time in decades since the sequencing of GFP in 1990, left, to the present, right. POI, protein of interest; TF, transcription factor.

A complex blend of transgenic optical tools, the blue light-inducible TEV protease (BLITz) system, combines the CRY2/CIB1 system with the AsLOV2 system to precisely induce transcription upon exposure to blue light [212] (Fig 4A). BLITz consists of 2 parts, a membrane-bound fusion of CIBN (a truncated version of CIB1), the N-terminal portion of the tobacco etch virus protease (TEV), AsLOV2, TEV cleavage site, and a transcriptional activator, and a soluble portion consisting of CRY2PHR (a truncated version of CRY2) and the C-terminus of TEV (C-TEV). Upon exposure to blue light, binding of CRY2PHR to CIBN facilitates interaction of the 2 parts of TEV while simultaneously exposing the TEV cleavage site, allowing cleavage and release of the transcriptional activator.

Another tool in the modern genetic engineer’s toolbox is the light-activated reversible inhibition by assembled trap (LARIAT) strategy [213]. LARIAT consists of a CIB1-bound CaMKIIα and a CRY2-bound POI. Since CaMKIIα self-oligomerizes, exposure to blue light reversibly traps the POI in clusters. The PixE/PixD homo/heterodimer scheme has also been used to conditionally induce/dissociate subcellular protein droplets in the PixELLs system [219]. In PixELLs, blue light exposure diffuses phase-separated droplets formed in the dark through a combination of the liquid–liquid phase separation function of N-terminal intrinsically disordered protein region (IDR) of the human FUS protein (FUS^N^) [214] and the oligomerization of PixE/PixD under dark conditions (see Fig 3J). A similar mechanism to PixELLs, developed in the same study, is optoDroplets which uses CRY2. In optoDroplets, FUSN is fused to CRY2 to form phase separated droplets when blue light is applied to promote oligomerization of CRY2 [215].

LANSTRAP and CLASP both improve upon the LANS system for LOV2-mediated nuclear import [216,217] (see Fig 4B). LANSTRAP uses a membrane-bound Zdk2 to directly bind LANS in the dark, whereas CLASP uses a membrane-bound LOV2 to trap a Zdk2:POI:LANS fusion in the dark, thereby improving nuclear exclusion of LANS pre-blue light exposure. On the other hand, LINXnano [157] fuses LINX to nano, a truncated version of SspB, which facilitates binding to a membrane-bound iLID in blue light (see Fig 4C). This improvement of the LINX system produces more complete nuclear export by tethering exported LINXnano:POIs within the cytoplasm. Similar to LANSTRAP and CLASP in function is iRIS, which sequesters the LOV2:NLS-fused POI in the cytoplasm via fusion to Q-PAS1 which facilitates binding to a membrane-anchored BphP1 when exposed to far-red light and nuclear import by NLS exposure when exposed to blue light [218].

Specific Protein Association tool giving transcriptional Readout with rapid Kinetics (SPARK) is a tool devised to conditionally report cells in which 2 POIs are interacting [219]. One POI is fused to a TEV protease while the other is fused to a modified LOV2:TEV cleavage site:TF. Upon exposure to blue light, the TEV cleavage site is exposed allowing for release of the TF and activation of reporter gene(s) in cells where the 2 POIs are interacting.

Cyanobacteriochrome-based light-inducible dimers (BICYCL) employs a modified light-induced isomerizing GAF (cGMP-specific phosphodies-terases, adenylyl cyclases and FhlA) domain derived from *Acaryochloris marina* AM1_C0023g2, Amg2, to shift binding between 2 binding partners when exposed to either red or green light [220]. Binding partners, binder of Amg2-red state (BAmRed) and binder of Amg2-red state (BAmGreen), were engineered such that Amg2 binds BAmRed when exposed to red light and to BAmGreen when exposed to green light, allowing for POI swapping via light exposure (Fig 4D). The developers of BICYCL showed that it can be used to conditionally recruit either a transcriptional repressor or activator to DNA-tethered Amg2 depending on laser color choice.

Blue-OFF combines LOV2-mediated transcriptional inhibition with psd-mediated POI degradation to more completely eliminate POI presence when exposed to blue light [221] (Fig 4E). LOV2 is used to create a cryptic DNA-binding domain and is further fused to a transcriptional inhibitor (KRAB) to eliminate transcription of the POI which is fused to B-LID for degron-mediated depletion. Analogously, iLight controls gene transcription via far red mediated homodimerization of the photosensory core module of *Idiomarina* IsPadC (IsPadC-PMC) fused to either LexA408 to block transcription or Gal4 and VP16 to trigger gene expression, which can be reversed via exposure to near-infrared light [222] (Fig 4F). In the dark or after exposure to near-infrared light, the fused TFs are sterically inhibited by isomeric transformations of IsPadC-PMC.

## Discussion

While there have been many reviews on optical tagging systems, optical biosensors, optogenetics, and biologically expressed imaging tools more broadly [223], we have focused this compendium specifically on tools currently available for use in transgenic plant and animal studies. In a few decades, molecular biology and genetic engineering fields have exploded from complex transgenics being a hypothetical potentiality to thousands of transgenic organisms, hundreds of fluorescent proteins and tags, scores of subcellular sensors, and dozens of applications for several optogenetic systems.

It is important to carefully consider the limitations of each class of optical tool as well as for each individual instrument therein. For example, labeling with one of the tag systems described here comes with the disadvantage of occupying a laser and detector on the microscope merely to label, so it’s sometimes prudent to use a sensor also as a label which may come with the disadvantages of slow fluorescent maturation and low signal. Similarly, within classes of optical tools, different tools with similar functions have important advantages and limitations to consider. For example, degrons require transcription and translation of replacement fusion POIs to recover after depletion while translocation tools require mere shuttling within the cell to recover; however, degrons more quickly and completely deplete POI levels. Further, it’s necessary to contemplate the activation/inactivation times of optogenetic tools which often take a couple to tens of minutes to reach peak activation as well as their efficiencies of activity and reversibility which tend to land all over the map. In addition, when considering the use of sensors or tags in combination with optogenetic tools, it’s imperative to weigh the cost in photobleaching and phototoxicity against the reward of maintained optogenetic activation and tailor experiments accordingly. As discussed in the previous section, combinatorial applications of these tools are being developed which synergistically supplement each individual component’s limitations.

It is expected that we will soon see a surge in development of sophisticated tools and studies monitoring chromatin architecture, transcription, translation, protein localization, and cellular responses via reporters, tags, and sensors while simultaneously manipulating transcription or protein levels or function in living transgenic plants and animals. One such study has already reached preprint from the lab of Hernan Garcia manipulating transcription factors via LEXY while simultaneously monitoring transcription of their targets via MS2/MCP [224]. Another ongoing study from the lab of Sanjeevi Sivasankar seeks to combine optogenetics and BioID by fusing CRY2 or CIB1 to 2 halves of a split-TurboID biotinylation enzyme thereby allowing for precise temporal control of the enzyme’s activity [225].

**Fig 5:**
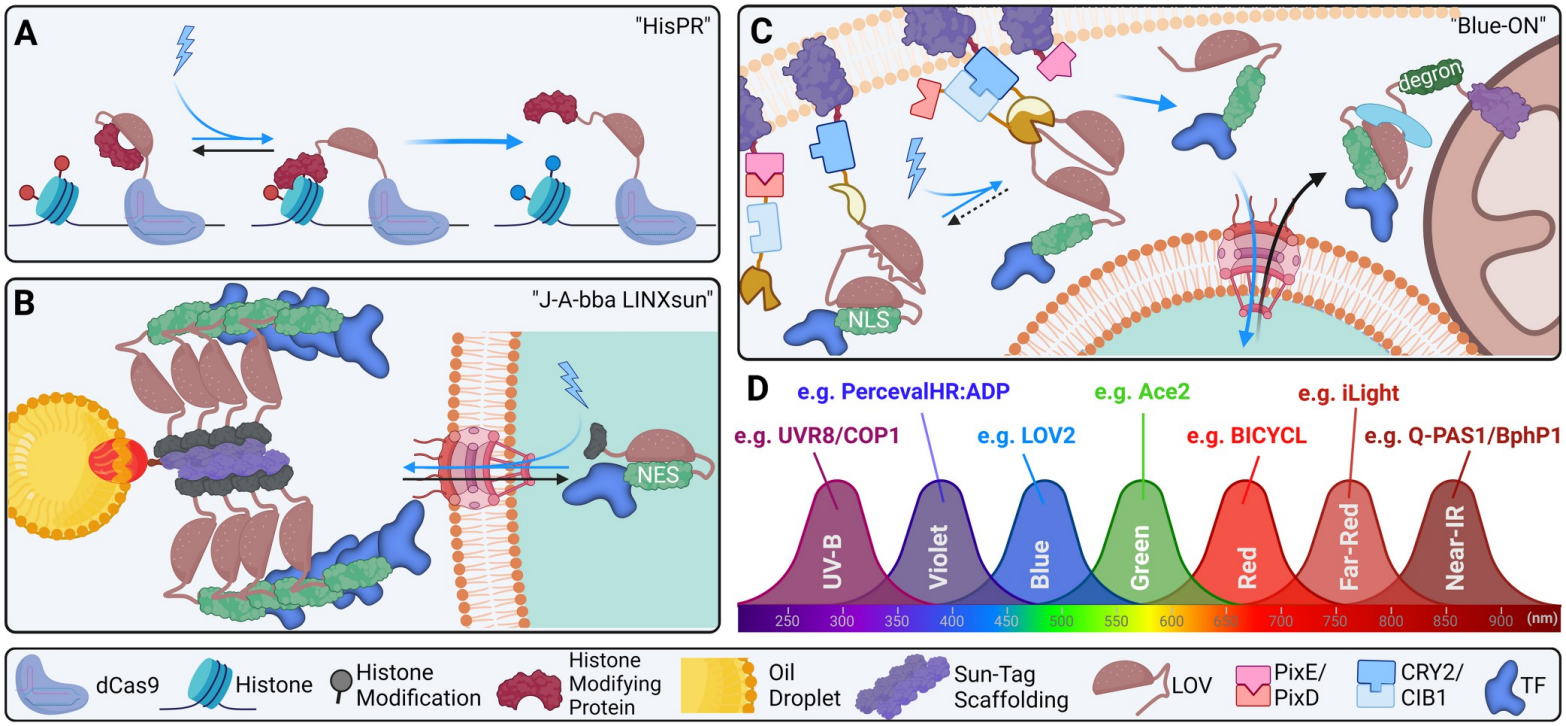
Future directions of transgenic optical tools. (A) Histone modification by CRISPR (“HisPR”): LOV2 can hypothetically be used to conditionally expose a cryptic histone modifying enzyme targeted to genomic loci by dCAS9. (B) Jabba-trapped LINX via SunTag (“J-A-bba LINXsun”): Jabba-trap was developed to trap fusion proteins on lipid droplets dispersed throughout the pre-cellularization *Drosophila* embryo [226]. In conjunction with LINX and SunTag, it may be possible to precisely trigger nuclear export of TFs via blue light until gastrulation begins. (C) “Blue-ON”: Blue light releases a membrane-caged caspase fragment via PixE/PixD action. Caspase fragments assemble in similar fashion to BLITZ under blue light exposure via iLID/SspB action to release an NLS-fused nuclear factor via caspase recognition sequence exposure by LOV2. LANS:TF fusion is uptaken by the nucleus while exposed to blue light as in LANS. In the dark, the LANS construct is exported from the nucleus via NES, sequesters at mitochondrial-bound Zdk, and is degraded via fused degron sequence. While quite complex, this system would produce near complete silencing of overexpression models in the dark; further, once a transgenic is produced in one species, only the TF needs be replaced to produce tools for any other target. (D) Currently, it may be possible to use up to 7 transgenic optical tools simultaneously. LANS, light-activated nuclear shuttle; TF, transcription factor.

Additionally, we have conceived of a few hypothetical advancements which we believe can be useful if developed. First, precise spatiotemporal control of epigenetic states has the potential to be an invaluable tool for interrogating development and disease and can feasibly be accomplished by AsLOV2-toggling of histone modifiers and genomic targeting via gRNAs and dCAS9 (see Fig 5A). Second, combining tags with other systems is sure to soon gain popularity; using SUN-tag to sequester transcription factors conditioned upon pre-cellularization conditions in the early *Drosophila* embryo, e.g., could be useful for specific enquiries into early development (see Fig 5B). Third, the BLITZ system can feasibly be modified to control overexpressed transgenic transcription factors which would otherwise accumulate in systems like LINXnano and confound expectations of spatiotemporal precision (see Fig 5C).

Nontoxic blue and red light are certainly most useful for in vivo studies in transgenic animals, complex systems encorporating varieties of optogenetic tools will require multiple lasers to be used in concert. It is exciting that several systems have already been developed which implement bohemian wavelengths for activation, including far-red, near-infrared, violet, and UV-B (see Fig 5D). With appropriate experimental design and controls, it is hypothetically possible to simultaneously use a handful of different lasers to affect different optical tools and achieve increasingly complex objectives.

We will undoubtedly see further exponential growth of the field of transgenic optical tool development and application in the coming years. Neurological and metabolic disorders stand to benefit greatly from future advances which will undoubtedly include translational applications with medical potential. Encouragingly, advances in ex utero [227–229] and in utero [230,231] experimental techniques and technologies promise to bring optogenetics to mammalian embryonic development, an important step toward this goal. Further, the genetically encoded devices described herein offer our best chance at supplementing our insufficient understanding of basic cell, developmental, and disease mechanisms which have hitherto remained inaccessible. This review is meant to be a brief almanac of the tools available to those who will develop and use these future models and devices.

## References

[1] Bouwman BAM, Crosetto N, Bienko M. The era of 3D and spatial genomics. Trends Genet. 2022 Oct 1;38(10):1062–75. doi: 10.1016/j.tig.2022.05.010 35680466

[2] Soroczynski J, Risca VI. Technological advances in probing 4D genome organization. Curr Opin Cell Biol. 2023 Oct 1;84:102211. doi: 10.1016/j.ceb.2023.102211 37556867 PMC10588670

[3] Van Gijtenbeek LA, Kok J. Illuminating Messengers: An Update and Outlook on RNA Visualization in Bacteria. Front Microbiol. 2017;8:1161. doi: 10.3389/fmicb.2017.01161 28690601 PMC5479882

[4] Asp M, Bergenstråhle J, Lundeberg J. Spatially Resolved Transcriptomes—Next Generation Tools for Tissue Exploration. Bioessays. 2020;42(10):1–16. doi: 10.1002/bies.201900221 32363691

[5] Ding J, Sharon N, Bar-Joseph Z. Temporal modelling using single-cell transcriptomics [Internet]. Vol. 23, Nature Reviews Genetics. Nature Publishing Group; 2022 [cited 2023 Sep 7]. p. 355–68. Available from: https://www.nature.com/articles/s41576-021-00444-7. doi: 10.1038/s41576-021-00444-7 35102309 PMC10354343

[6] Tian L, Chen F, Macosko EZ. The expanding vistas of spatial transcriptomics [Internet]. Vol. 41, Nature Biotechnology. Nature Publishing Group; 2023 [cited 2023 Sep 7]. p. 773–82. Available from: https://www.nature.com/articles/s41587-022-01448-2. doi: 10.1038/s41587-022-01448-2 36192637 PMC10091579

[7] Cui M, Cheng C, Zhang L. High-throughput proteomics: a methodological mini-review [Internet]. Vol. 102, Laboratory Investigation. Nature Publishing Group; 2022 [cited 2023 Sep 7]. p. 1170–81. Available from: https://www.nature.com/articles/s41374-022-00830-7. doi: 10.1038/s41374-022-00830-7 35922478 PMC9362039

[8] Bennett HM, Stephenson W, Rose CM, Darmanis S. Single-cell proteomics enabled by next-generation sequencing or mass spectrometry [Internet]. Vol. 20, Nature Methods. Nature Publishing Group; 2023 [cited 2023 Sep 7]. p. 363–74. Available frem: https://www.nature.com/articles/s41592-023-01791-5. 10.1038/s41592-023-01791-5 36864196

[9] Vandereyken K, Sifrim A, Thienpont B, Voet T. Methods and applications for single-cell and spatial multi-omics [Internet]. Vol. 24, Nature Reviews Genetics. Nature Publishing Group; 2023 [cited 2023 Sep 7]. p. 494–515. Available from: https://www.nature.com/articles/s41576-023-00580-2. doi: 10.1038/s41576-023-00580-2 36864178 PMC9979144

[10] Brodbelt JS. Deciphering combinatorial post-translational modifications by top-down mass spectrometry [Internet]. Vol. 70, Current Opinion in Chemical Biology. Annual Reviews; 2022 [cited 2023 Sep 7]. p. 157–79. Available from: https://www.annualreviews.org/doi/abs/10.1146/annurev-biophys-092721-085421. doi: 10.1016/j.cbpa.2022.102180 35779351 PMC9489649

[11] Karch KR, Snyder DT, Harvey SR, Wysocki VH. Native Mass Spectrometry: Recent Progress and Remaining Challenges [Internet]. Vol. 51, Annual Review of Biophysics Annual Reviews; 2022 [cited 2023 Sep 7]. p. 157–79. Available from: https://www.annualreviews.org/doi/abs/10.1146/annurev-biophys-092721-085421. 34982572 10.1146/annurev-biophys-092721-085421 PMC10700022

[12] Kang MG, Rhee HW. Molecular Spatiomics by Proximity Labeling. Acc Chem Res [Internet]. 2022 [cited 2023 Sep 7];2022:37. Available from: https://pubs.acs.org/doi/full/10.1021/acs.accounts.2c00061. doi: 10.1021/acs.accounts.2c00061 35512328 PMC9118551

[13] Suzuki Y, Kadomatsu K, Sakamoto K. Towards the in vivo identification of protein-protein interactions. J Biochem [Internet]. 2023 May 29 [cited 2023 Sep 7];173(6):413–5. doi: 10.1093/jb/mvad013 36821413

[14] Shkel O, Kharkivska Y, Kim YK, Lee JS. Proximity Labeling Techniques: A Multi-Omics Toolbox. Chem Asian J. 2022 Jan 17;17(2). doi: 10.1002/asia.202101240 34850572

[15] Prasher DC, Eckenrode VK, Ward WW, Prendergast FG, Cormier MJ. Primary structure of the Aequorea victoria green-fluorescent protein. Gene. 1992 Feb 15;111(2):229–33. doi: 10.1016/0378-1119(92)90691-h 1347277

[16] Riani YD, Matsuda T, Takemoto K, Nagai T. Green monomeric photosensitizing fluorescent protein for photo-inducible protein inactivation and cell ablation. BMC Biol [Internet]. 2018 Apr 30 [cited 2023 Aug 16];16(1):1–12. Available from: https://link.springer.com/articles/10.1186/s12915-018-0514-7. 29712573 10.1186/s12915-018-0514-7 PMC5928576

[17] Nakai J, Ohkura M, Imoto K. A high signal-to-noise Ca2+ probe composed of a single green fluorescent protein. Nat Biotechnol [Internet]. 2001;19:137–141. Available from: http://biotech.nature.com. doi: 10.1038/84397 11175727

[18] Bothma JP, Norstad MR, Alamos S, Garcia HG. LlamaTags: A Versatile Tool to Image Transcription Factor Dynamics in Live Embryos. Cell [Internet]. 2018 [cited 2023 Jul 18];173(7):1810–1822.e16. doi: 10.1016/j.cell.2018.03.069 29754814 PMC6003873

[19] Toran P, Smolina I, Driscoll H, Ding F, Sun Y, Cantor CR, et al. Labeling native bacterial RNA in live cells. Cell Res. 2014;24(7):894–897. doi: 10.1038/cr.2014.47 24732010 PMC4085757

[20] Marques SM, Slanska M, Chmelova K, Chaloupkova R, Marek M, Clark S, et al. Mechanism-Based Strategy for Optimizing HaloTag Protein Labeling. JACS Au [Internet]. 2022 [cited 2023 Jul 19];2(6):1324–37. doi: 10.1021/jacsau.2c00002 35783171 PMC9241015

[21] Tanenbaum ME, Gilbert LA, Qi LS, Weissman JS, Vale RD. A protein tagging system for signal amplification in gene expression and fluorescence imaging. Cell [Internet]. 2014 Oct 10 [cited 2023 Aug 10];159(3):635. doi: 10.1016/j.cell.2014.09.039 25307933 PMC4252608

[22] Harmansa S, Affolter M. Protein binders and their applications in developmental biology. Development (Cambridge) [Internet]. 2018 Jan 15 [cited 2023 Sep 6];145(2). doi: 10.1242/dev.148874 29374062

[23] Takemoto K, Matsuda T, Sakai N, Fu D, Noda M, Uchiyama S, et al. SuperNova, a monomeric photosensitizing fluorescent protein for chromophore-assisted light inactivation. Sci Rep [Internet]. 2013 Sep 17 [cited 2023 Sep 6];3(1):1–7. Available from: https://www.nature.com/articles/srep02629. doi: 10.1038/srep02629 24043132 PMC3775092

[24] De Meyer T, Muyldermans S, Depicker A. Nanobody-based products as research and diagnostic tools. Trends Biotechnol [Internet]. 2014 May 1 [cited 2023 Oct 2];32(5):263–70. Available from: http://www.cell.com/article/S0167779914000419/fulltext. doi: 10.1016/j.tibtech.2014.03.001 24698358

[25] Xu J, Kim AR, Cheloha RW, Fischer FA, Li JSS, Feng Y, et al. Protein visualization and manipulation in Drosophila through the use of epitope tags recognized by nanobodies. Elife. 2022 Jan 1;11. doi: 10.7554/eLife.74326 35076390 PMC8853664

[26] Yiu HW, Demidov VV, Toran P, Cantor CR, Broude NE. RNA detection in live bacterial cells using fluorescent protein complementation triggered by interaction of two RNA aptamers with two RNA-binding peptides. Pharmaceuticals. 2011;4(3):494–508.

[27] van Gijtenbeek LA, Kok J. Illuminating messengers: An update and outlook on RNA visualization in bacteria. Front Microbiol [Internet]. 2017 [cited 2023 Jul 10];8(JUN):1–19. Available from: www.frontiersin.org. doi: 10.3389/fmicb.2017.01161 28690601 PMC5479882

[28] Bertrand E, Chartrand P, Schaefer M, Shenoy SM, Singer RH, Long RM. Localization of ASH1 mRNA particles in living yeast. Mol Cell. 1998;2(4):437–445. doi: 10.1016/s1097-2765(00)80143-4 9809065

[29] Tutucci E, Vera M, Biswas J, Garcia J, Parker R, Singer RH. An improved ms2 system for accurate reporting of the mrnA life cycle. 2018;15(1). 10.1038/nmeth.4502 PMC5843578 29131164

[30] Lim F, Peabody DS. RNA recognition site of PP7 coat protein. Nucleic Acids Res [Internet]. 2002 Oct 10 [cited 2023 Sep 6];30(19):4138. doi: 10.1093/nar/gkf552 12364592 PMC140551

[31] Heinrich S, Sidler CL, Azzalin CM, Weis K. Stem-loop RNA labeling can affect nuclear and cytoplasmic mRNA processing. RNA [Internet]. 2017 Feb 1 [cited 2023 Sep 6];23(2):134–41. doi: 10.1261/rna.057786.116 28096443 PMC5238788

[32] Fukaya T, Lim B, Levine M. Rapid Rates of Pol II Elongation in the Drosophila Embryo. Curr Biol. 2017 May 8;27(9):1387–91. doi: 10.1016/j.cub.2017.03.069 28457866 PMC5665007

[33] Levo M, Raimundo J, Bing XY, Sisco Z, Batut PJ, Ryabichko S, et al. Transcriptional coupling of distant regulatory genes in living embryos. Nature [Internet]. 2022 May 4 [cited 2023 Aug 14];605(7911):754–60. Available from: https://www.nature.com/articles/s41586-022-04680-7. doi: 10.1038/s41586-022-04680-7 35508662 PMC9886134

[34] Viushkov VS, Lomov NA, Rubtsov MA, Vassetzky YS. Visualizing the Genome: Experimental Approaches for Live-Cell Chromatin Imaging [Internet]. Vol. 11, Cells. Multidisciplinary Digital Publishing Institute; 2022 [cited 2023 Aug 14]. p. 4086. Available from: https://www.mdpi.com/2073-4409/11/24/4086/htm. doi: 10.3390/cells11244086 36552850 PMC9776900

[35] Vinter DJ, Hoppe C, Ashe HL. Live and fixed imaging of translation sites at single mRNA resolution in the Drosophila embryo. STAR Protoc [Internet]. 2021 [cited 2023 Jul 18];2(3):100812. doi: 10.1016/j.xpro.2021.100812 34585149 PMC8450298

[36] Koromila T, Stathopoulos A. Distinct Roles of Broadly Expressed Repressors Support Dynamic Enhancer Action and Change in Time. Cell Rep [Internet]. 2019;28(4):855–863.e5. doi: 10.1016/j.celrep.2019.06.063 31340149 PMC6927530

[37] Koromila T, Gao F, Iwasaki Y, He P, Pachter L, Peter Gergen J, et al. Odd-paired is a pioneer-like factor that coordinates with zelda to control gene expression in embryos. Elife. 2020 Jul 1;9:1–71. doi: 10.7554/eLife.59610 32701060 PMC7417190

[38] Ohishi H, Shimada S, Uchino S, Li J, Sato Y, Shintani M, et al. STREAMING-tag system reveals spatiotemporal relationships between transcriptional regulatory factors and transcriptional activity. Nat Commun [Internet]. 2022 Dec 20 [cited 2023 Oct 10];13(1):1–19. Available from: https://www.nature.com/articles/s41467-022-35286-2. 36539402 10.1038/s41467-022-35286-2 PMC9768169

[39] Birnie A, Plat A, Korkmaz C, Bothma JP. Precisely timed regulation of enhancer activity defines the binary expression pattern of Fushi tarazu in the Drosophila embryo. Curr Biol. 2023 Jul 24;33(14):2839–2850.e7. doi: 10.1016/j.cub.2023.04.005 37116484 PMC10373528

[40] Chen J, Nikolaitchik O, Singh J, Wright A, Bencsics CE, Coffin JM, et al. High efficiency of HIV-1 genomic RNA packaging and heterozygote formation revealed by single virion analysis. Proc Natl Acad Sci U S A. 2009 Aug 11;106(32):13535–40. doi: 10.1073/pnas.0906822106 19628694 PMC2714765

[41] Takizawa PA, Vale RD. The myosin motor, Myo4p, binds Ash1 mRNA via the adapter protein, She3p. Proc Natl Acad Sci U S A [Internet]. 2000 May 9 [cited 2023 Sep 6];97(10):5273–8. doi: 10.1073/pnas.080585897 10792032 PMC25818

[42] Schönberger J, Hammes UZ, Dresselhaus T. In vivo visualization of RNA in plants cells using the λN22 system and a GATEWAY-compatible vector series for candidate RNAs. Plant J [Internet]. 2012 Jul 1 [cited 2023 Sep 6];71(1):173–81. doi: 10.1111/j.1365-313X.2012.04923.x 22268772

[43] Ye J, Silverman L, Lairmore MD, Green PL. HTLV-1 Rex is required for viral spread and persistence in vivo but is dispensable for cellular immortalization in vitro. Blood [Internet]. 2003 Dec 12 [cited 2023 Sep 6];102(12):3963. doi: 10.1182/blood-2003-05-1490 12907436 PMC2852248

[44] Das AT, Harwig A, Berkhout B. The HIV-1 Tat Protein Has a Versatile Role in Activating Viral Transcription. J Virol [Internet]. 2011 Sep 15 [cited 2023 Sep 6];85(18):9506. doi: 10.1128/JVI.00650-11 21752913 PMC3165771

[45] Rentmeister A, Mannack LVJC, Eising S. Current techniques for visualizing RNA in cells. F1000Res [Internet]. 2016 [cited 2023 Sep 6];5. 10.12688/f1000research.8151.1 PMC4850879 27158473

[46] Ozawa T, Natori Y, Sato M, Umezawa Y. Imaging dynamics of endogenous mitochondrial RNA in single living cells. Nat Methods. 2007;4(5):413–419. doi: 10.1038/nmeth1030 17401370

[47] Suzuki C, Garces RG, Edmonds KA, Hiller S, Hyberts SG, Wagner AM. PDCD4 inhibits translation initiation by binding to eIF4A using both its MA3 domains. Proc Natl Acad Sci U S A [Internet]. 2008 Mar 4 [cited 2023 Sep 6];105(9):3274–9. doi: 10.1073/pnas.0712235105 18296639 PMC2265113

[48] Feng S, Sekine S, Pessino V, Li H, Leonetti MD, Huang B. Improved split fluorescent proteins for endogenous protein labeling. Nat Commun [Internet]. 2017 Aug 29 [cited 2023 Sep 7];8(1):1–11. Available from: https://www.nature.com/articles/s41467-017-00494-8. 28851864 10.1038/s41467-017-00494-8 PMC5575300

[49] Furman JL, Badran AH, Shen S, Stains CI, Hannallah J, Segal DJ, et al. Systematic evaluation of split-fluorescent proteins for the direct detection of native and methylated DNA. Bioorg Med Chem Lett. 2009 Jul 15;19(14):3748–51. doi: 10.1016/j.bmcl.2009.04.141 19457665 PMC2709697

[50] Wang X, McLachlan J, Zamore PD, Hall TMT. Modular recognition of RNA by a human Pumilio-homology domain. Cell [Internet]. 2002 Aug 23 [cited 2023 Aug 14];110(4):501–12. Available from: http://www.cell.com/article/S0092867402008735/fulltext. doi: 10.1016/s0092-8674(02)00873-5 12202039

[51] Tilsner J, Linnik O, Christensen NM, Bell K, Roberts IM, Lacomme C, et al. Live-cell imaging of viral RNA genomes using a Pumilio-based reporter. Plant J [Internet]. 2009 Feb 1 [cited 2023 Aug 14];57(4):758–70. doi: 10.1111/j.1365-313X.2008.03720.x 18980643

[52] Adamala KP, Martin-Alarcon DA, Boydena ES. Programmable RNA-binding protein composed of repeats of a single modular unit. Proc Natl Acad Sci U S A [Internet]. 2016 May 10 [cited 2023 Sep 7];113(19):E2579–88. doi: 10.1073/pnas.1519368113 27118836 PMC4868411

[53] Chen B, Gilbert LA, Cimini BA, Schnitzbauer J, Zhang W, Li GW, et al. Dynamic imaging of genomic loci in living human cells by an optimized CRISPR/Cas system. Cell [Internet]. 2013 Dec 19 [cited 2023 Oct 10];155(7):1479–91. Available from: http://www.cell.com/article/S0092867413015316/fulltext. doi: 10.1016/j.cell.2013.12.001 24360272 PMC3918502

[54] Lindhout BI, Fransz P, Tessadori F, Meckel T, Hooykaas PJJ, van der Zaal BJ. Live cell imaging of repetitive DNA sequences via GFP-tagged polydactyl zinc finger proteins. Nucleic Acids Res [Internet]. 2007 Aug 15 [cited 2023 Oct 10];35(16):e107–e107. doi: 10.1093/nar/gkm618 17704126 PMC2018617

[55] Borisov SM, Wolfbeis OS. Optical biosensors. Chem Rev. 2008 Feb;108(2):423–61. doi: 10.1021/cr068105t 18229952

[56] Wang H, Jing M, Li Y. Lighting up the brain: genetically encoded fluorescent sensors for imaging neurotransmitters and neuromodulators. Curr Opin Neurobiol. 2018 Jun 1;50:171–8. doi: 10.1016/j.conb.2018.03.010 29627516 PMC5984720

[57] Cole NB, Smith CL, Sciaky N, Terasaki M, Edidin M, Lippincott-Schwartz J. Diffusional Mobility of Golgi Proteins in Membranes of Living Cells. Science (1979). 1996;273(5276):797–801. doi: 10.1126/science.273.5276.797 8670420

[58] Zhang Y, Avalos JL. Traditional and novel tools to probe the mitochondrial metabolism in health and disease. Wiley Interdiscip Rev Syst Biol Med. 2017;9(2):1373. doi: 10.1002/wsbm.1373 28067471

[59] Wang M, Da Y, Tian Y. Fluorescent proteins and genetically encoded biosensors. Chem Soc Rev. 2023 Feb 20;52(4):1189–214. doi: 10.1039/d2cs00419d 36722390

[60] Hochreiter B, Garcia AP, Schmid JA. Fluorescent proteins as genetically encoded FRET biosensors in life sciences. Sensors (Switzerland). 2015;15(10):26281–26314. doi: 10.3390/s151026281 26501285 PMC4634415

[61] Wu L, Huang C, Emery BP, Sedgwick AC, Bull SD, He XP, et al. Förster resonance energy transfer (FRET)-based small-molecule sensors and imaging agents. Chem Soc Rev. 2020;49:5110–5139. 32697225 10.1039/c9cs00318e PMC7408345

[62] Llères D, James J, Swift S, Norman DG, Lamond AI. Quantitative analysis of chromatin compaction in living cells using FLIM-FRET. J Cell Biol. 2009;187(4):481–496. doi: 10.1083/jcb.200907029 19948497 PMC2779238

[63] Day RN. Visualization of Pit-1 Transcription Factor Interactions in the Living Cell Nucleus by Fluorescence Resonance Energy Transfer Microscopy. Mol Endocrinol. 1998 Sep 1;12(9):1410–9. doi: 10.1210/mend.12.9.0168 9731708

[64] Dupont S, Wickström SA. Mechanical regulation of chromatin and transcription. Vol. 23, Nature Reviews Genetics. Nature Publishing Group; 2022. p. 624–43. doi: 10.1038/s41576-022-00493-6 35606569

[65] Fenelon KD, Hopyan S. Structural components of nuclear integrity with gene regulatory potential. Curr Opin Cell Biol. 2017;48:63–71. doi: 10.1016/j.ceb.2017.06.001 28641117

[66] Wang C, Yang J. Mechanical forces: The missing link between idiopathic pulmonary fibrosis and lung cancer. Eur J Cell Biol. 2022 Jun 1;101(3):151234. doi: 10.1016/j.ejcb.2022.151234 35569385

[67] Vignes H, Vagena-Pantoula C, Prakash M, Fukui H, Norden C, Mochizuki N, et al. Extracellular mechanical forces drive endocardial cell volume decrease during zebrafish cardiac valve morphogenesis. Dev Cell. 2022;57(5):598–609.e5. doi: 10.1016/j.devcel.2022.02.011 35245444

[68] Zuela-Sopilniak N, Lammerding J. Can’t handle the stress? Mechanobiology and disease. Trends Mol Med. 2022 Sep 1;28(9):710–25. doi: 10.1016/j.molmed.2022.05.010 35717527 PMC9420767

[69] Sanfeliu-Cerdán N, Lin LC, Dunn AR, Goodman MB, Krieg M. Visualizing Neurons Under Tension In Vivo with Optogenetic Molecular Force Sensors. Methods Mol Biol. 2023:239–266. doi: 10.1007/978-1-0716-2851-5_16 36587102 PMC11874908

[70] Maurer M, Lammerding J. The Driving Force: Nuclear Mechanotransduction in Cellular Function, Fate, and Disease. Annu Rev Biomed Eng. 2019;21:443–468. doi: 10.1146/annurev-bioeng-060418-052139 30916994 PMC6815102

[71] Alisafaei F, Jokhun DS, Shivashankar GV, Shenoy VB. Regulation of nuclear architecture, mechanics, and nucleocytoplasmic shuttling of epigenetic factors by cell geometric constraints. Proc Natl Acad Sci U S A. 2019;116(27):13200–13209. doi: 10.1073/pnas.1902035116 31209017 PMC6613080

[72] Yang C, Zhang X, Guo Y, Meng F, Sachs F, Guo J. Mechanical dynamics in live cells and fluorescence-based force/tension sensors. Biochim Biophys Acta Mol Cell Res. 2015;1853(8):1889–1904. doi: 10.1016/j.bbamcr.2015.05.001 25958335 PMC4841255

[73] Grashoff C, Hoffman BD, Brenner MD, Zhou R, Parsons M, Yang MT, et al. Measuring mechanical tension across vinculin reveals regulation of focal adhesion dynamics. Nature. 2010 Jul 8;466(7303):263–6. doi: 10.1038/nature09198 20613844 PMC2901888

[74] Carley E, Stewart RK, Zieman A, Jalilian I, King DE, Zubek A, et al. The linc complex transmits integrin-dependent tension to the nuclear lamina and represses epidermal differentiation. Elife. 2021 Mar 1;10. 10.7554/eLife.58541 PMC8051949 33779546

[75] Fenelon KD, Thomas E, Samani M, Zhu M, Tao H, Sun Y, et al. Transgenic force sensors and software to measure force transmission across the mammalian nuclear envelope in vivo. Biol Open. 2022;11(11). doi: 10.1242/bio.059656 36350289 PMC9672859

[76] Guo J, Sachs F, Meng F. Fluorescence-based force/tension sensors: A novel tool to visualize mechanical forces in structural proteins in live cells. Antioxid Redox Signal. 2014;20(6):986–999. doi: 10.1089/ars.2013.5708 24205787 PMC3924807

[77] Jin X, Rosenbohm J, Minnick G, Esfahani AM, Safa BT, Yang R. Cell characterization by nanonewton force sensing. In: Robotics for Cell Manipulation and Characterization. Academic Press; 2023. p. 245–70.

[78] Das R, Lin LC, Català-Castro F, Malaiwong N, Sanfeliu-Cerdán N, Porta-De-la-Riva M, et al. An asymmetric mechanical code ciphers curvature-dependent proprioceptor activity. Sci Adv. 2021 Sep 1;7(38):4617–34. doi: 10.1126/sciadv.abg4617 34533987 PMC8448456

[79] Brenner MD, Zhou R, Conway DE, Lanzano L, Gratton E, Schwartz MA, et al. Spider Silk Peptide Is a Compact, Linear Nanospring Ideal for Intracellular Tension Sensing. Nano Lett. 2016 Mar 9;16(3):2096–102. doi: 10.1021/acs.nanolett.6b00305 26824190 PMC4851340

[80] Cost AL, Ringer P, Chrostek-Grashoff A, Grashoff C. How to Measure Molecular Forces in Cells: A Guide to Evaluating Genetically-Encoded FRET-Based Tension Sensors. Cell Mol Bioeng. 2015;8(1):96–105. doi: 10.1007/s12195-014-0368-1 25798203 PMC4361753

[81] Gadella TWJ. Fret and Flim Techniques. van der Vliet PC, Pillai S, editors. Elsevier; 2009. p. 1–534.

[82] Ishikawa-Ankerhold HC, Ankerhold R, Drummen GPC. Advanced fluorescence microscopy techniques-FRAP, FLIP, FLAP, FRET and FLIM. Molecules. 2012;17(4):4047–4132. doi: 10.3390/molecules17044047 22469598 PMC6268795

[83] Borghi N, Sorokina M, Shcherbakova OG, Weis WI, Pruitt BL, Nelson WJ, et al. E-cadherin is under constitutive actomyosin-generated tension that is increased at cell-cell contacts upon externally applied stretch. Proc Natl Acad Sci U S A. 2012;109(31):12568–12573. doi: 10.1073/pnas.1204390109 22802638 PMC3411997

[84] Haas AJ, Zihni C, Ruppel A, Hartmann C, Ebnet K, Tada M, et al. Interplay between Extracellular Matrix Stiffness and JAM-A Regulates Mechanical Load on ZO-1 and Tight Junction Assembly. Cell Rep. 2020 Jul 21;32(3):107924. doi: 10.1016/j.celrep.2020.107924 32697990 PMC7383227

[85] Tao H, Zhu M, Lau K, Whitley OKW, Samani M, Xiao X, et al. Oscillatory cortical forces promote three dimensional cell intercalations that shape the murine mandibular arch. Nat Commun. 2019 Apr 12;10(1):1–18. 30979871 10.1038/s41467-019-09540-z PMC6461694

[86] Arsenovic PT, Ramachandran I, Bathula K, Zhu R, Narang JD, Noll NA, et al. Nesprin-2G, a Component of the Nuclear LINC Complex, Is Subject to Myosin-Dependent Tension. Biophys J [Internet]. 2016;110(1):34–43. doi: 10.1016/j.bpj.2015.11.014 26745407 PMC4805861

[87] Kannan M, Vasan G, Huang C, Haziza S, Li JZ, Inan H, et al. Fast, in vivo voltage imaging using a red fluorescent indicator. Nat Methods. 2018 Nov 12;15(12):1108–16. doi: 10.1038/s41592-018-0188-7 30420685 PMC6516062

[88] Gong Y, Huang C, Li JZ, Grewe BF, Zhang Y, Eismann S, et al. High-speed recording of neural spikes in awake mice and flies with a fluorescent voltage sensor. Science (1979) [Internet]. 2015 Dec 11 [cited 2023 Aug 17];350(6266):1361–6. Available from: www.sciencemag.org/content/350/6266/1357/suppl/DC1. doi: 10.1126/science.aab0810 26586188 PMC4904846

[89] Potzkei J, Kunze M, Drepper T, Gensch T, Jaeger KE, Büchs J. Real-time determination of intracellular oxygen in bacteria using a genetically encoded FRET-based biosensor. BMC Biol. 2012 Mar 22;10(1). doi: 10.1186/1741-7007-10-28 22439625 PMC3364895

[90] Papkovsky DB, Dmitriev RI. Biological detection by optical oxygen sensing. Chem Soc Rev. 2013;42:8700. doi: 10.1039/c3cs60131e 23775387

[91] Tyas L, Brophy VA, Pope A, Rivett AJ, Tavaré JM. Rapid caspase-3 activation during apoptosis revealed using fluorescence-resonance energy transfer. EMBO Rep [Internet]. 2000 Sep 1 [cited 2023 Sep 11];1(3):266–70. doi: 10.1093/embo-reports/kvd050 11256610 PMC1083724

[92] Suzuki M, Shindo Y, Yamanaka R, Oka K. Live imaging of apoptotic signaling flow using tunable combinatorial FRET-based bioprobes for cell population analysis of caspase cascades. Sci Rep [Internet]. 2022 Dec 7 [cited 2023 Sep 11];12(1):1–12. Available from: https://www.nature.com/articles/s41598-022-25286-z. 36476686 10.1038/s41598-022-25286-z PMC9729311

[93] Wu X, Simone J, Hewgill D, Siegel R, Lipsky PE, He L. Measurement of two caspase activities simultaneously in living cells by a novel dual FRET fluorescent indicator probe. Cytometry A. 2006 Jun;69(6):477–86. doi: 10.1002/cyto.a.20300 16683263

[94] Takemoto K, Nagai T, Miyawaki A, Miura M. Spatio-temporal activation of caspase revealed by indicator that is insensitive to environmental effects. J Cell Biol [Internet]. 2003 Jan 20 [cited 2023 Sep 11];160(2):235–43. doi: 10.1083/jcb.200207111 12527749 PMC2172647

[95] Bozza WP, Di X, Takeda K, R Rosado LA, Pariser S, Zhang B. The Use of a Stably Expressed FRET Biosensor for Determining the Potency of Cancer Drugs. PLoS ONE [Internet]. 2014 [cited 2023 Sep 11];9(9):e107010. Available from: https://journals.plos.org/plosone/article?id=10.1371/journal.pone.0107010. 25188024 10.1371/journal.pone.0107010 PMC4154796

[96] Onuki R, Nagasaki A, Kawasaki H, Baba T, Uyeda TQP, Taira K. Confirmation by FRET in individual living cells of the absence of significant amyloid β-mediated caspase 8 activation. Proc Natl Acad Sci U S A [Internet]. 2002 Nov 12 [cited 2023 Sep 13];99(23):14716–21. Available from: https://www.pnas.org/doi/abs/10.1073/pnas.232177599. 12409609 10.1073/pnas.232177599 PMC137485

[97] Kominami K, Nagai T, Sawasaki T, Tsujimura Y, Yashima K, Sunaga Y, et al. In Vivo Imaging of Hierarchical Spatiotemporal Activation of Caspase-8 during Apoptosis. PLoS ONE [Internet]. 2012 Nov 21 [cited 2023 Sep 13];7(11):e50218. Available from: https://journals.plos.org/plosone/article?id=10.1371/journal.pone.0050218. 23185580 10.1371/journal.pone.0050218 PMC3503975

[98] Ng S, Lim HS, Ma Q, Gao Z. Optical aptasensors for adenosine triphosphate. Theranostics. 2016;6(10):1683–1702. doi: 10.7150/thno.15850 27446501 PMC4955066

[99] Imamura H, Huynh Nhat KP, Togawa H, Saito K, Iino R, Kato-Yamada Y, et al. Visualization of ATP levels inside single living cells with fluorescence resonance energy transfer-based genetically encoded indicators. Proc Natl Acad Sci U S A. 2009 Sep 15;106(37):15651–6. doi: 10.1073/pnas.0904764106 19720993 PMC2735558

[100] Trevisiol A, Saab AS, Winkler U, Marx G, Imamura H, Möbius W, et al. Monitoring ATP dynamics in electrically active white matter tracts. Elife. 2017 Apr 17;6. doi: 10.7554/eLife.24241 28414271 PMC5415357

[101] Kishikawa JI, Fujikawa M, Imamura H, Yasuda K, Noji H, Ishii N, et al. MRT Letter: Expression of ATP Sensor Protein in Caenorhabditis elegans. Microsc Res Tech. 2012. doi: 10.1002/jemt.21103 22038755

[102] De Col V, Fuchs P, Nietzel T, Elsässer M, Voon CP, Candeo A, et al. ATP sensing in living plant cells reveals tissue gradients and stress dynamics of energy physiology. Elife. 2017 Jul 18;6. doi: 10.7554/eLife.26770 28716182 PMC5515573

[103] Prinz A, Diskar M, Herberg FW. Application of bioluminescence resonance energy transfer (BRET) for biomolecular interaction studies. Vol. 7, ChemBioChem. John Wiley & Sons, Ltd; 2006. p. 1007–12. doi: 10.1002/cbic.200600048 16755626

[104] Pfleger KDG, Eidne KA. Illuminating insights into protein-protein interactions using bioluminescence resonance energy transfer (BRET). Nat Methods. 2006;3:165–174. doi: 10.1038/nmeth841 16489332

[105] Eder D, Basler K, Aegerter CM. Challenging FRET-based E-Cadherin force measurements in Drosophila. Sci Rep. 2017;7(1). doi: 10.1038/s41598-017-14136-y 29057959 PMC5651909

[106] Boute N, Jockers R, Issad T. The use of resonance energy transfer in high-throughput screening: BRET versus FRET. Trends Pharmacol Sci. 2002 Aug 1;23(8):351–4. doi: 10.1016/s0165-6147(02)02062-x 12377570

[107] Wu Y, Jiang T. Developments in FRET- and BRET-Based Biosensors [Internet]. Vol. 13, Micromachines. Multidisciplinary Digital Publishing Institute; 2022 [cited 2023 Aug 17]. p. 1789. Available from: https://www.mdpi.com/2072-666X/13/10/1789/htm. doi: 10.3390/mi13101789 36296141 PMC9610962

[108] Erdenee E, Ting AY. A Dual-Purpose Real-Time Indicator and Transcriptional Integrator for Calcium Detection in Living Cells. ACS Synth Biol [Internet]. 2022 Mar 18 [cited 2023 Aug 22];11(3):1086–95. doi: 10.1021/acssynbio.1c00597 35254056 PMC10395047

[109] Valkovic AL, Leckey MB, Whitehead AR, Hossain MA, Inoue A, Kocan M, et al. Real-time examination of cAMP activity at relaxin family peptide receptors using a BRET-based biosensor. Pharmacol Res Perspect. 2018 Oct 1;6(5). doi: 10.1002/prp2.432 30263124 PMC6153321

[110] Jiang LI, Collins J, Davis R, Lin KM, DeCamp D, Roach T, et al. Use of a cAMP BRET Sensor to Characterize a Novel Regulation of cAMP by the Sphingosine 1-Phosphate/G13 Pathway. J Biol Chem. 2007 Apr 6;282(14):10576–84. doi: 10.1074/jbc.M609695200 17283075 PMC2526465

[111] Ramirez MP, Anderson MJM, Kelly MD, Sundby LJ, Hagerty AR, Wenthe SJ, et al. Dystrophin missense mutations alter focal adhesion tension and mechanotransduction. Proc Natl Acad Sci U S A [Internet]. 2022 Jun 21 [cited 2023 Jul 24];119(25):e2205536119. Available from: https://www.pnas.org/doi/abs/10.1073/pnas.2205536119. 35700360 10.1073/pnas.2205536119 PMC9231619

[112] Oshino R, Oshino N, Tamura M, Kobilinsky L, Chance B. A sensitive bacterial luminescence probe for O2 in biochemical systems. Biochim Biophys Acta. 1972 Jun 26;273(1):5–17. 5038288 10.1016/0304-4165(72)90185-7

[113] Den Hamer A, Dierickx P, Arts R, De Vries JSPM, Brunsveld L, Merkx M. Bright Bioluminescent BRET Sensor Proteins for Measuring Intracellular Caspase Activity. ACS Sens [Internet]. 2017 Jun 23 [cited 2023 Aug 13];2(6):729–34. Available from: https://pubs.acs.org/sharingguidelines. doi: 10.1021/acssensors.7b00239 28670623 PMC5485374

[114] Xu Y, Piston DW, Johnson CH. A bioluminescence resonance energy transfer (BRET) system: Application to interacting circadian clock proteins. Proc Natl Acad Sci U S A [Internet]. 1999 Jan 5 [cited 2023 Aug 13];96(1):151–6. doi: 10.1073/pnas.96.1.151 9874787 PMC15108

[115] Wu Y, Jiang T. Developments in FRET- and BRET-Based Biosensors. Vol. 13, Micromachines. Multidisciplinary Digital Publishing Institute; 2022. p. 1789. doi: 10.3390/mi13101789 36296141 PMC9610962

[116] Khakhar A, Starker CG, Chamness JC, Lee N, Stokke S, Wang C, et al. Building customizable auto-luminescent luciferase-based reporters in plants. Elife. 2020 Mar 1;9. 10.7554/eLife.52786 PMC7164954 32209230

[117] Mitiouchkina T, Mishin AS, Somermeyer LG, Markina NM, Chepurnyh TV, Guglya EB, et al. Plants with genetically encoded autoluminescence. Nat Biotechnol. 2020;38(8):944–946. doi: 10.1038/s41587-020-0500-9 32341562 PMC7610436

[118] Müller K, Engesser R, Timmer J, Zurbriggen MD, Nagy F, Weber W. Synthesis of phycocyanobilin in mammalian cells. Chem Commun. 2013 Sep 10;49(79):8970–2. doi: 10.1039/c3cc45065a 23963496

[119] Zhou Y, Kong D, Wang X, Yu G, Wu X, Guan N, et al. A small and highly sensitive red/far-red optogenetic switch for applications in mammals. Nat Biotechnol. 2022 Oct 4;40(2):262–72. doi: 10.1038/s41587-021-01036-w 34608325

[120] Rodriguez-Garcia A, Rojo-Ruiz J, Navas-Navarro P, Aulestia FJ, Gallego-Sandin S, Garcia-Sancho J, et al. GAP, an aequorin-based fluorescent indicator for imaging Ca2+ in organelles. Proc Natl Acad Sci U S A. 2014 Feb 18;111(7):2584–9. doi: 10.1073/pnas.1316539111 24501126 PMC3932923

[121] Yang C, Zhang X, Guo Y, Meng F, Sachs F, Guo J. Mechanical dynamics in live cells and fluorescence-based force/tension sensors. Biochim Biophys Acta Mol Cell Res [Internet]. 2015 [cited 2023 Jul 24];1853(8):1889–904. doi: 10.1016/j.bbamcr.2015.05.001 25958335 PMC4841255

[122] Baird GS, Zacharias DA, Tsien RY. Circular permutation and receptor insertion within green fluorescent proteins. Proc Natl Acad Sci U S A. 1999 Sep 28;96(20):11241–6. doi: 10.1073/pnas.96.20.11241 10500161 PMC18018

[123] Topell S, Hennecke J, Glockshuber R. Circularly permuted variants of the green fluorescent protein. FEBS Lett. 1999 Aug 27;457(2):283–9. doi: 10.1016/s0014-5793(99)01044-3 10471794

[124] Iwai S, Uyeda TQP. Visualizing myosin-actin interaction with a genetically-encoded fluorescent strain sensor. Proc Natl Acad Sci U S A. 2008 Nov 4;105(44):16882–7. doi: 10.1073/pnas.0805513105 18971336 PMC2579347

[125] De Angelis DA, Miesenböck G, Zemelman BV, Rothman JE. PRIM: Proximity imaging of green fluorescent protein-tagged polypeptides. Proc Natl Acad Sci U S A. 1998 Oct 13;95(21):12312–6. doi: 10.1073/pnas.95.21.12312 9770483 PMC22828

[126] Tian L, Hires SA, Mao T, Huber D, Chiappe ME, Chalasani SH, et al. Imaging neural activity in worms, flies and mice with improved GCaMP calcium indicators. Nat Methods. 2009;6(12):875–881. doi: 10.1038/nmeth.1398 19898485 PMC2858873

[127] Berg J, Hung YP, Yellen G. A genetically encoded fluorescent reporter of ATP/ADP ratio. Nat Methods. 2009;6(2):161. doi: 10.1038/nmeth.1288 19122669 PMC2633436

[128] Li J, Yu Q, Ahooghalandari P, Gribble FM, Reimann F, Tengholm A, et al. Submembrane ATP and Ca2+ kinetics in α-cells: Unexpected signaling for glucagon secretion. FASEB J. 2015 Aug 1;29(8):3379–88. 25911612 10.1096/fj.14-265918 PMC4539996

[129] Tantama M, Martínez-François JR, Mongeon R, Yellen G. Imaging energy status in live cells with a fluorescent biosensor of the intracellular ATP-to-ADP ratio. Nat Commun [Internet]. 2013 [cited 2023 Jul 29];4. Available from: www.nature.com/naturecommunications. doi: 10.1038/ncomms3550 24096541 PMC3852917

[130] Diuba AV, Samigullin DV, Kaszas A, Zonfrillo F, Malkov A, Petukhova E, et al. CLARITY analysis of the Cl/pH sensor expression in the brain of transgenic mice. Neuroscience. 2020 Jul 15;439:181–94. doi: 10.1016/j.neuroscience.2019.07.010 31302264

[131] Batti L, Mukhtarov M, Audero E, Ivanov A, Paolicelli O, Zurborg S, et al. Transgenic mouse lines for non-invasive ratiometric monitoring of intracellular chloride. Front Mol Neurosci. 2013 May 21;6(MAY):44911. doi: 10.3389/fnmol.2013.00011 23734096 PMC3659292

[132] Fischer AAM, Kramer MM, Radziwill G, Weber W. Shedding light on current trends in molecular optogenetics. Curr Opin Chem Biol. 2022 Oct 1;70. doi: 10.1016/j.cbpa.2022.102196 35988347

[133] Kichuk TC, Carrasco-López C, Avalos JL, José Avalos CL. Lights up on organelles: Optogenetic tools to control subcellular structure and organization. WIREs Mech Dis. 2020. doi: 10.1002/wsbm.1500 32715616

[134] Chia N, Lee SY, Tong Y. Optogenetic tools for microbial synthetic biology. Biotechnol Adv. 2022 Oct 1;59. doi: 10.1016/j.biotechadv.2022.107953 35398205

[135] Labella ML, Sigrist S, Jørgensen EM. Putting genetics into optogenetics: Knocking out proteins with light. In: Optogenetics. 2013:79–90.

[136] Ledri M, Andersson M, Wickham J, Kokaia M. Optogenetics for controlling seizure circuits for translational approaches. Neurobiol Dis. 2023 Aug 1;184:106234. doi: 10.1016/j.nbd.2023.106234 37479090

[137] Usherenko S, Stibbe H, Muscò M, Essen LO, Kostina EA, Taxis C. Photo-sensitive degron variants for tuning protein stability by light. BMC Syst Biol [Internet]. 2014 Nov 18 [cited 2023 Jul 20];8(1):128. doi: 10.1186/s12918-014-0128-9 25403319 PMC4236813

[138] Usherenko S, Stibbe H, Muscò M, Essen LO, Kostina EA, Taxis C. Photo-sensitive degron variants for tuning protein stability by light. BMC Syst Biol. 2014 Nov 18;8(1):128. doi: 10.1186/s12918-014-0128-9 25403319 PMC4236813

[139] Renicke C, Schuster D, Usherenko S, Essen LO, Taxis C. A LOV2 domain-based optogenetic tool to control protein degradation and cellular function. Chem Biol. 2013;20(4):619–626. doi: 10.1016/j.chembiol.2013.03.005 23601651

[140] Bonger KM, Rakhit R, Payumo AY, Chen JK, Wandless TJ. General method for regulating protein stability with light. ACS Chem Biol. 2014 Jan 17;9(1):111–5. doi: 10.1021/cb400755b 24180414 PMC3906921

[141] Bonger KM, Chen LC, Liu CW, Wandless TJ. Small-molecule displacement of a cryptic degron causes conditional protein degradation. Nat Chem Biol. 2011;7(8):531–537. doi: 10.1038/nchembio.598 21725303 PMC3139708

[142] Stevens LM, Kim G, Koromila T, Steele JW, McGehee J, Stathopoulos A, et al. Light-dependent N-end rule-mediated disruption of protein function in Saccharomyces cerevisiae and Drosophila melanogaster. PLoS Genet. 2021 May 17;17(5):e1009544. doi: 10.1371/journal.pgen.1009544 33999957 PMC8158876

[143] Varshavsky A. N-degron and C-degron pathways of protein degradation. Proc Natl Acad Sci U S A. 2019 Jan 8;116(2):358–66. doi: 10.1073/pnas.1816596116 30622213 PMC6329975

[144] Heo AJ, Bin KS, Kwon YT, Ji CH. The N-degron pathway: From basic science to therapeutic applications. Vol. 1866, Biochimica et Biophysica Acta—Gene Regulatory Mechanisms. Elsevier; 2023. p. 194934. doi: 10.1016/j.bbagrm.2023.194934 36990317

[145] Ryu KA, Kaszuba CM, Bissonnette NB, Oslund RC, Fadeyi OO. Interrogating biological systems using visible-light-powered catalysis. Vol. 5, Nature Reviews Chemistry. Nature Publishing Group; 2021. p. 322–37. doi: 10.1038/s41570-021-00265-6 37117838

[146] Takemoto K, Sekiya T. Optical manipulation of molecular function by chromophore-assisted light inactivation. Proc Jpn Acad Ser B. 2021 Apr 9;97(4):197–209. doi: 10.2183/pjab.97.011 33840676 PMC8062263

[147] Liao JC, Roider J, Jay DG. Chromophore-assisted laser inactivation of proteins is mediated by the photogeneration of free radicals. Proc Natl Acad Sci U S A. 1994 Mar 29;91(7):2659–63. doi: 10.1073/pnas.91.7.2659 8146171 PMC43429

[148] Bulina ME, Chudakov DM, Britanova OV, Yanushevich YG, Staroverov DB, Chepurnykh TV, et al. A genetically encoded photosensitizer. Nat Biotechnol. 2006 Dec 20;24(1):95–9. doi: 10.1038/nbt1175 16369538

[149] Riani YD, Matsuda T, Takemoto K, Nagai T. Green monomeric photosensitizing fluorescent protein for photo-inducible protein inactivation and cell ablation. BMC Biol. 2018 Apr 30;16(1):1–12. 29712573 10.1186/s12915-018-0514-7 PMC5928576

[150] Takemoto K, Matsuda T, Sakai N, Fu D, Noda M, Uchiyama S, et al. SuperNova, a monomeric photosensitizing fluorescent protein for chromophore-assisted light inactivation. Sci Rep. 2013 Sep 17;3(1):1–7. doi: 10.1038/srep02629 24043132 PMC3775092

[151] Duffy JB. GAL4 system in Drosophila: A fly geneticist’s Swiss army knife. Genesis. 2002;34(1–2):1–15. doi: 10.1002/gene.10150 12324939

[152] Sadowski I, Ma J, Triezenberg S, Ptashne M. GAL4-VP16 is an unusually potent transcriptional activator. Nature. 1988;335(6190):563–564. doi: 10.1038/335563a0 3047590

[153] Zhu L, McNamara HM, Toettcher JE. Light-switchable transcription factors obtained by direct screening in mammalian cells. Nat Commun. 2023 Jun 2;14(1):1–16. 37268649 10.1038/s41467-023-38993-6 PMC10238501

[154] Yumerefendi H, Dickinson DJ, Wang H, Zimmerman SP, Bear JE, Goldstein B, et al. Control of protein activity and cell fate specification via light-mediated nuclear translocation. PLoS ONE. 2015;10(6):1–19. doi: 10.1371/journal.pone.0128443 26083500 PMC4471001

[155] Niopek D, Benzinger D, Roensch J, Draebing T, Wehler P, Eils R, et al. Engineering light-inducible nuclear localization signals for precise spatiotemporal control of protein dynamics in living cells. 2014 Jul 14;5(1). 10.1038/ncomms5404 PMC4104460 25019686

[156] Lerner AM, Yumerefendi H, Goudy OJ, Strahl BD, Kuhlman B. Engineering Improved Photoswitches for the Control of Nucleocytoplasmic Distribution. ACS Synth Biol [Internet]. 2018 Nov 15 [cited 2023 Sep 7];7(12):2898–907. doi: 10.1021/acssynbio.8b00368 30441907 PMC6497050

[157] Yumerefendi H, Lerner AM, Parker Zimmerman S, Hahn K, Bear JE, Strahl BD, et al. Light-induced nuclear export reveals rapid dynamics of epigenetic modifications. Nat Chem Biol [Internet]. 2016 [cited 2023 Aug 14];12. Available from: www.nature.com/naturechemicalbiology. doi: 10.1038/nchembio.2068 27089030 PMC4888063

[158] Kögler AC, Kherdjemil Y, Bender K, Rabinowitz A, Marco-Ferreres R, Furlong EEM. Extremely rapid and reversible optogenetic perturbation of nuclear proteins in living embryos. Dev Cell [Internet]. 2021;56(16):2348–2363.e8. Available from: https://www.sciencedirect.com/science/article/pii/S1534580721005943. doi: 10.1016/j.devcel.2021.07.011 34363757 PMC8387026

[159] Niopek D, Wehler P, Roensch J, Eils R, Di Ventura B. Optogenetic control of nuclear protein export. Nat Commun. 2016;7:1–9. doi: 10.1038/ncomms10624 26853913 PMC4748110

[160] Qiao J, Peng H, Dong B. Development and Application of an Optogenetic Manipulation System to Suppress Actomyosin Activity in Ciona Epidermis. Int J Mol Sci. 2023;24(6). doi: 10.3390/ijms24065707 36982781 PMC10054466

[161] Berlew EE, Kuznetsov IA, Yamada K, Bugaj LJ, Boerckel JD, Chow BY. Single-Component Optogenetic Tools for Inducible RhoA GTPase Signaling. Adv Biol. 2021 Sep 1;5(9):2100810. doi: 10.1002/adbi.202100810 34288599 PMC8446339

[162] Hannanta-Anan P, Glantz ST, Chow BY. Optically inducible membrane recruitment and signaling systems. Vol. 57, Current Opinion in Structural Biology. 2019. p. 84–92. doi: 10.1016/j.sbi.2019.01.017 30884362 PMC6697567

[163] Berlew EE, Kuznetsov IA, Yamada K, Bugaj LJ, Chow BY. Optogenetic Rac1 engineered from membrane lipid-binding RGS-LOV for inducible lamellipodia formation. Photochem Photobiol Sci. 2020 Mar 1;19(3):353–61. doi: 10.1039/c9pp00434c 32048687 PMC7141788

[164] Nash AI, McNulty R, Shillito ME, Swartz TE, Bogomolni RA, Luecke H, et al. Structural basis of photosensitivity in a bacterial light-oxygen-voltage/ helix-turn-helix (LOV-HTH) DNA-binding protein. Proc Natl Acad Sci U S A. 2011 Jun 7;108(23):9449–54. doi: 10.1073/pnas.1100262108 21606338 PMC3111320

[165] Rivera-Cancel G, Motta-Mena LB, Gardner KH. Identification of natural and artificial DNA substrates for light-activated LOV-HTH transcription factor EL222. Biochemistry. 2012 Dec 18;51(50):10024–34. doi: 10.1021/bi301306t 23205774 PMC3531242

[166] Gligorovski V, Sadeghi A, Rahi SJ. Multidimensional characterization of inducible promoters and a highly light-sensitive LOV-transcription factor. Nat Commun. 2023 Jun 27;14(1):1–18. 37369667 10.1038/s41467-023-38959-8 PMC10300134

[167] Wang Z, Yan Y, Zhang H. Design and Characterization of an Optogenetic System in Pichia pastoris. ACS Synth Biol. 2022 Jan 21;11(1):297–307. doi: 10.1021/acssynbio.1c00422 34994189

[168] Zhao EM, Lalwani MA, Lovelett RJ, Garciá-Echauri SA, Hoffman SM, Gonzalez CL, et al. Design and Characterization of Rapid Optogenetic Circuits for Dynamic Control in Yeast Metabolic Engineering. ACS Synth Biol. 2020 Dec 18;9(12):3254–66. doi: 10.1021/acssynbio.0c00305 33232598 PMC10399620

[169] Zhao EM, Lalwani MA, Chen JM, Orillac P, Toettcher JE, Avalos JL. Optogenetic Amplification Circuits for Light-Induced Metabolic Control. ACS Synth Biol. 2021 May 21;10(5):1143–54. doi: 10.1021/acssynbio.0c00642 33835777 PMC8721662

[170] Zhao EM, Zhang Y, Mehl J, Park H, Lalwani MA, Toettcher JE, et al. Optogenetic regulation of engineered cellular metabolism for microbial chemical production. Nature. 2018 Mar 21;555(7698):683–7. doi: 10.1038/nature26141 29562237 PMC5876151

[171] Pathak GP, Strickland D, Vrana JD, Tucker CL. Benchmarking of optical dimerizer systems. ACS Synth Biol. 2014 Nov 21;3(11):832–8. doi: 10.1021/sb500291r 25350266 PMC4277767

[172] Guntas G, Hallett RA, Zimmerman SP, Williams T, Yumerefendi H, Bear JE, et al. Engineering an improved light-induced dimer (iLID) for controlling the localization and activity of signaling proteins. Proc Natl Acad Sci U S A. 2015 Jan 6;112(1):112–7. doi: 10.1073/pnas.1417910112 25535392 PMC4291625

[173] Lungu OI, Hallett RA, Choi EJ, Aiken MJ, Hahn KM, Kuhlman B. Designing Photoswitchable Peptides Using the AsLOV2 Domain. Chem Biol. 2012 Apr 20;19(4):507–17. doi: 10.1016/j.chembiol.2012.02.006 22520757 PMC3334866

[174] Keenan SE, Blythe SA, Marmion RA, Djabrayan NJV, Wieschaus EF, Shvartsman SY. Rapid Dynamics of Signal-Dependent Transcriptional Repression by Capicua. Dev Cell. 2020 Mar 23;52(6):794–801.e4. doi: 10.1016/j.devcel.2020.02.004 32142631 PMC7161736

[175] Johnson HE, Goyal Y, Pannucci NL, Schüpbach T, Shvartsman SY, Toettcher JE. The Spatiotemporal Limits of Developmental Erk Signaling. Dev Cell. 2017 Jan 23;40(2):185–92. doi: 10.1016/j.devcel.2016.12.002 28118601 PMC5289754

[176] Goglia AG, Wilson MZ, Jena SG, Silbert J, Basta LP, Devenport D, et al. A Live-Cell Screen for Altered Erk Dynamics Reveals Principles of Proliferative Control. Cell Syst. 2020 Mar 25;10(3):240–253.e6. doi: 10.1016/j.cels.2020.02.005 32191874 PMC7540725

[177] Farahani PE, Lemke SB, Dine E, Uribe G, Toettcher JE, Nelson CM. Substratum stiffness regulates Erk signaling dynamics through receptor-level control. Cell Rep. 2021;37(13). doi: 10.1016/j.celrep.2021.110181 34965432 PMC8756379

[178] Johnson HE. Application of Optogenetics to Probe the Signaling Dynamics of Cell Fate Decision-Making. In: Computational Modeling of Signaling Networks. Springer; 2023. p. 315–26. 10.1007/978-1-0716-3008-2_14 37074585

[179] Kennedy MJ, Hughes RM, Peteya LA, Schwartz JW, Ehlers MD, Tucker CL. Rapid blue-light-mediated induction of protein interactions in living cells. Nat Methods. 2010;7(12):973–975. doi: 10.1038/nmeth.1524 21037589 PMC3059133

[180] Bugaj LJ, Choksi AT, Mesuda CK, Kane RS, Schaffer D V. Optogenetic protein clustering and signaling activation in mammalian cells. Nat Methods. 2013 Feb 3;10(3):249–52. doi: 10.1038/nmeth.2360 23377377

[181] Singh AP, Wu P, Ryabichko S, Raimundo J, Swan M, Wieschaus E, et al. Optogenetic control of the Bicoid morphogen reveals fast and slow modes of gap gene regulation. Cell Rep. 2022 Mar 22;38(12):110543. doi: 10.1016/j.celrep.2022.110543 35320726 PMC9019726

[182] McDaniel SL, Gibson TJ, Schulz KN, Fernandez Garcia M, Nevil M, Jain SU, et al. Continued Activity of the Pioneer Factor Zelda Is Required to Drive Zygotic Genome Activation. Mol Cell. 2019 Apr 4;74(1):185–195.e4. doi: 10.1016/j.molcel.2019.01.014 30797686 PMC6544384

[183] Crefcoeur RP, Yin R, Ulm R, Halazonetis TD. Ultraviolet-B-mediated induction of protein-protein interactions in mammalian cells. Nat Commun. 2013 Apr 30;4(1):1–7. doi: 10.1038/ncomms2800 23653191

[184] Yang X, Jost APT, Weiner OD, Tang C. A light-inducible organelle-targeting system for dynamically activating and inactivating signaling in budding yeast. Mol Biol Cell. 2013 Aug 1;24(15):2419–30. doi: 10.1091/mbc.E13-03-0126 23761071 PMC3727934

[185] Toettcher JE, Weiner OD, Lim WA. Using Optogenetics to Interrogate the Dynamic Control of Signal Transmission by the Ras/Erk Module. Cell. 2013 Dec 5;155(6):1422–34. doi: 10.1016/j.cell.2013.11.004 24315106 PMC3925772

[186] Lan TH, He L, Huang Y, Zhou Y. Optogenetics for transcriptional programming and genetic engineering. Trends Genet [Internet]. 2022 Dec 1 [cited 2023 Sep 6];38(12):1253–70. Available from: http://www.cell.com/article/S0168952522001408/fulltext. doi: 10.1016/j.tig.2022.05.014 35738948 PMC10484296

[187] Braatsch S, Johnson JA, Noll K, Beatty JT. The O2-responsive repressor PpsR2 but not PpsR1 transduces a light signal sensed by the BphP1 phytochrome in Rhodopseudomonas palustris CGA009. FEMS Microbiol Lett. 2007 Jul 1;272(1):60–4. doi: 10.1111/j.1574-6968.2007.00734.x 17456182

[188] Redchuk TA, Kaberniuk AA, Verkhusha VV. Near-infrared light-controlled systems for gene transcription regulation, protein targeting and spectral multiplexing. Nat Protoc. 2018 Apr 26;13(5):1121–36. doi: 10.1038/nprot.2018.022 29700485 PMC6574219

[189] Redchuk TA, Omelina ES, Chernov KG, Verkhusha V V. Near-infrared optogenetic pair for protein regulation and spectral multiplexing. Nat Chem Biol. 2017 Mar 27;13(6):633–9. doi: 10.1038/nchembio.2343 28346403 PMC6239862

[190] Benedetti L, Marvin JS, Falahati H, Guillén-Samander A, Looger LL, De Camilli P. Optimized vivid-derived magnets photodimerizers for subcellular optogenetics in mammalian cells. Elife. 2020;9:1–49. doi: 10.7554/eLife.63230 33174843 PMC7735757

[191] Kawano F, Suzuki H, Furuya A, Sato M. Engineered pairs of distinct photoswitches for optogenetic control of cellular proteins. Nat Commun. 2015 Feb 24;6(1):1–8. doi: 10.1038/ncomms7256 25708714

[192] Zoltowski BD, Crane BR. Light activation of the LOV protein vivid generates a rapidly exchanging dimer. Biochemistry. 2008 Jul 8;47(27):7012–9. doi: 10.1021/bi8007017 18553928 PMC2743001

[193] Zoltowski BD, Schwerdtfeger C, Widom J, Loros JJ, Bilwes AM, Dunlap JC, et al. Conformational switching in the fungal light sensor vivid. Science (1979). 2007 May 18;316(5827):1054–7. doi: 10.1126/science.1137128 17510367 PMC3682417

[194] Vaidya AT, Chen CH, Dunlap JC, Loros JJ, Crane BR. Structure of a light-activated LOV protein dimer that regulates transcription. Sci Signal. 2011 Aug 2;4(184). doi: 10.1126/scisignal.2001945 21868352 PMC3401549

[195] Wang X, Chen X, Yang Y. Spatiotemporal control of gene expression by a light-switchable transgene system. Nat Methods. 2012;9(3):266–269. doi: 10.1038/nmeth.1892 22327833

[196] Yuan H, Bauer CE. PixE promotes dark oligomerization of the BLUF photoreceptor PixD. Proc Natl Acad Sci U S A. 2008 Aug 19;105(33):11715–9. doi: 10.1073/pnas.0802149105 18695243 PMC2575306

[197] Stierl M, Stumpf P, Udwari D, Gueta R, Hagedorn R, Losi A, et al. Light modulation of cellular cAMP by a small bacterial photoactivated adenylyl cyclase, bPAC, of the soil bacterium Beggiatoa. J Biol Chem. 2011;286(2):1181–1188. doi: 10.1074/jbc.M110.185496 21030594 PMC3020725

[198] Wang H, Vilela M, Winkler A, Tarnawski M, Schlichting I, Yumerefendi H, et al. LOVTRAP: An optogenetic system for photoinduced protein dissociation. Nat Methods. 2016 Jul 18;13(9):755–8. doi: 10.1038/nmeth.3926 27427858 PMC5137947

[199] Strickland D, Lin Y, Wagner E, Hope CM, Zayner J, Antoniou C, et al. TULIPs: tunable, light-controlled interacting protein tags for cell biology. Nat Methods [Internet]. 2012 Apr 4;9(4):379–84. Available from: https://www.nature.com/articles/nmeth.1904. doi: 10.1038/nmeth.1904 22388287 PMC3444151

[200] Huang J, Koide A, Makabe K, Koide S. Design of protein function leaps by directed domain interface evolution. Proc Natl Acad Sci U S A [Internet]. 2008 May 6 [cited 2023 Sep 13];105(18):6578–83. Available from: www.pnas.orgcgidoi10.1073pnas.0801097105. 18445649 10.1073/pnas.0801097105 PMC2373342

[201] Zhang W, Lohman AW, Zhuravlova Y, Lu X, Wiens MD, Hoi H, et al. Optogenetic control with a photocleavable protein, Phocl. Nat Methods. 2017 Mar 13;14(4):391–4. doi: 10.1038/nmeth.4222 28288123

[202] Kato HE, Kamiya M, Sugo S, Ito J, Taniguchi R, Orito A, et al. Atomistic design of microbial opsin-based blue-shifted optogenetics tools. Nat Commun [Internet]. 2015 May 15 [cited 2023 Sep 7];6(1):1–10. Available from: https://www.nature.com/articles/ncomms8177. doi: 10.1038/ncomms8177 25975962 PMC4479019

[203] Rost BR, Schneider F, Grauel MK, Wozny C, Bentz C, Blessing A, et al. Optogenetic acidification of synaptic vesicles and lysosomes. Nat Neurosci. 2015 Dec 9;18(12):1845–52. doi: 10.1038/nn.4161 26551543 PMC4869830

[204] Miesenböck G, De Angelis DA, Rothman JE. Visualizing secretion and synaptic transmission with pH-sensitive green fluorescent proteins. Nature. 1998 Dec 30;394(6689):192–5. doi: 10.1038/28190 9671304

[205] Shevchenko V, Mager T, Kovalev K, Polovinkin V, Alekseev A, Juettner J, et al. Inward H+ pump xenorhodopsin: Mechanism and alternative optogenetic approach. Sci Adv [Internet]. 2017 [cited 2023 Sep 11];3(9). doi: 10.1126/sciadv.1603187 28948217 PMC5609834

[206] Inoue K, Ono H, Abe-Yoshizumi R, Yoshizawa S, Ito H, Kogure K, et al. A light-driven sodium ion pump in marine bacteria. Nat Commun [Internet]. 2013 Apr 9 [cited 2023 Sep 11];4(1):1–10. Available from: https://www.nature.com/articles/ncomms2689. doi: 10.1038/ncomms2689 23575682

[207] Feroz H, Ferlez B, Lefoulon C, Ren T, Baker CS, Gajewski JP, et al. Light-Driven Chloride Transport Kinetics of Halorhodopsin. Biophys J [Internet]. 2018 Jul 7 [cited 2023 Sep 11];115(2):353. doi: 10.1016/j.bpj.2018.06.009 30021110 PMC6051019

[208] Govorunova EG, Sineshchekov OA, Li H, Wang Y, Brown LS, Spudich JL. RubyACRs, nonalgal anion channelrhodopsins with highly red-shifted absorption. Proc Natl Acad Sci U S A [Internet]. 2020 Sep 15 [cited 2023 Sep 13];117(37):22833–40. doi: 10.1073/pnas.2005981117 32873643 PMC7502701

[209] Scheib U, Stehfest K, Gee CE, Körschen HG, Fudim R, Oertner TG, et al. The rhodopsin-guanylyl cyclase of the aquatic fungus Blastocladiella emersonii enables fast optical control of cGMP signaling. Sci Signal [Internet]. 2015 [cited 2024 Feb 5];8(389). Available from: www.SCIENCESIGNALING.org. doi: 10.1126/scisignal.aab0611 26268609

[210] Yoshida K, Tsunoda SP, Brown LS, Kandori H. A unique choanoflagellate enzyme rhodopsin exhibits lightdependent cyclic nucleotide phosphodiesterase activity. J Biol Chem [Internet]. 2017 May 5 [cited 2024 Feb 5];292(18):7531–41. Available from: http://www.jbc.org/article/S0021925820428637/fulltext. doi: 10.1074/jbc.M117.775569 28302718 PMC5418051

[211] Gao S, Nagpal J, Schneider MW, Kozjak-Pavlovic V, Nagel G, Gottschalk A. Optogenetic manipulation of cGMP in cells and animals by the tightly light-regulated guanylyl-cyclase opsin CyclOp. Nat Commun [Internet]. 2015 [cited 2024 Feb 5];6. Available from: www.nature.com/naturecommunications. doi: 10.1038/ncomms9046 26345128 PMC4569695

[212] Lee D, Creed M, Jung K, Stefanelli T, Wendler DJ, Oh WC, et al. Temporally precise labeling and control of neuromodulatory circuits in the mammalian brain. Nat Methods. 2017 Apr 3;14(5):495–503. doi: 10.1038/nmeth.4234 28369042

[213] Lee S, Park H, Kyung T, Kim NY, Kim S, Kim J, et al. Reversible protein inactivation by optogenetic trapping in cells. Nat Methods. 2014;11(6):633–636. doi: 10.1038/nmeth.2940 24793453

[214] Patel A, Lee HO, Jawerth L, Maharana S, Jahnel M, Hein MY, et al. A Liquid-to-Solid Phase Transition of the ALS Protein FUS Accelerated by Disease Mutation. Cell. 2015 Aug 27;162(5):1066–77. doi: 10.1016/j.cell.2015.07.047 26317470

[215] Dine E, Gil AA, Uribe G, Brangwynne CP, Toettcher JE. Protein Phase Separation Provides Long-Term Memory of Transient Spatial Stimuli. Cell Syst. 2018 Jun 27;6(6):655–663.e5. doi: 10.1016/j.cels.2018.05.002 29859829 PMC6023754

[216] Yumerefendi H, Wang H, Ickinson DD, Erner AL, Malkus P, Goldstein B, et al. Light-Dependent Cytoplasmic Recruitment Enhances the Dynamic Range of a Nuclear Import Photoswitch. Chembiochem. 2018. doi: 10.1002/cbic.201700681 29446199 PMC6013380

[217] Chen SY, Osimiri LC, Chevalier M, Bugaj LJ, Nguyen TH, Greenstein RA, et al. Optogenetic Control Reveals Differential Promoter Interpretation of Transcription Factor Nuclear Translocation Dynamics. Cell Syst. 2020;11(4):336–353.e24. doi: 10.1016/j.cels.2020.08.009 32898473 PMC7648432

[218] Redchuk TA, Omelina ES, Chernov KG, Verkhusha VV. Near-infrared optogenetic pair for protein regulation and spectral multiplexing. Nat Chem Biol [Internet]. 2017 Mar 27 [cited 2023 Aug 26];13(6):633–9. Available from: https://www.nature.com/articles/nchembio.2343 doi: 10.1038/nchembio.2343 28346403 PMC6239862

[219] Kim MW, Wang W, Sanchez MI, Coukos R, von Zastrow M, Ting AY. Time-gated detection of protein-protein interactions with transcriptional readout. Elife. 2017;30:6. doi: 10.7554/eLife.30233 29189201 PMC5708895

[220] Jang J, Tang K, Youn J, McDonald S, Beyer HM, Zurbriggen MD, et al. Engineering of bidirectional, cyanobacteriochrome-based light-inducible dimers (BICYCL)s. Nat Methods [Internet]. 2023 Feb 23 [cited 2023 Aug 22];20(3):432–41. Available from: https://www.nature.com/articles/s41592-023-01764-8. doi: 10.1038/s41592-023-01764-8 36823330

[221] Baaske J, Gonschorek P, Engesser R, Dominguez-Monedero A, Raute K, Fischbach P, et al. Dual-controlled optogenetic system for the rapid down-regulation of protein levels in mammalian cells. Sci Rep. 2018 Oct 9;8(1):1–10. 30301909 10.1038/s41598-018-32929-7 PMC6177421

[222] Kaberniuk AA, Baloban M, Monakhov MV, Shcherbakova DM, Verkhusha VV. Single-component near-infrared optogenetic systems for gene transcription regulation. Nat Commun [Internet]. 2021 Jun 23 [cited 2023 Aug 16];12(1):1–12. Available from: https://www.nature.com/articles/s41467-021-24212-7. 34162879 10.1038/s41467-021-24212-7 PMC8222386

[223] Sittewelle M, Ferrandiz N, Fesenko M, Royle SJ. Genetically encoded imaging tools for investigating cell dynamics at a glance. J Cell Sci. 2023;136(7):1–7. doi: 10.1242/jcs.260783 37039102

[224] Zhao J, Lammers NC, Alamos S, Kim YJ, Martini G, Garcia HG. Optogenetic dissection of transcriptional repression in a multicellular organism. bioRxiv [Internet]. 2023 Apr 28 [cited 2023 Sep 7];2022.11.20.517211. Available from: https://www.biorxiv.org/content/10.1101/2022.11.20.517211v2. 10.1038/s41467-024-53539-0 PMC11513125 39461978

[225] Shafraz O, Marie C, Davis O, Sivasankar S. Light Activated BioID (LAB): an optically activated proximity labeling system to study protein-protein interactions. bioRxiv [Internet]. 2023 May 6 [cited 2023 Sep 7];2022.10.22.513249. doi: 10.1101/2022.10.22.513249v2 PMC10656424 37756605

[226] Seller CA, Cho CY, O’Farrell PH. Rapid embryonic cell cycles defer the establishment of heterochromatin by eggless/SetDB1 in Drosophila. Genes Dev [Internet]. 2019 Apr 1 [cited 2023 Aug 22];33(7–8):403–17. Available from: http://genesdev.cshlp.org/content/33/7-8/403.full. doi: 10.1101/gad.321646.118 30808658 PMC6446540

[227] Oldak B, Aguilera-Castrejon A, Hanna JH. Recent insights into mammalian natural and synthetic ex utero embryogenesis. Curr Opin Genet Dev. 2022;77:101988. doi: 10.1016/j.gde.2022.101988 36179582

[228] Neurovirulence of the Australian outbreak Japanese Encephalitis virus genotype 4 is lower. [cited 2024 Feb 5]. doi: 10.1101/2023.04.26.538504

[229] Aguilera-Castrejon A, Oldak B, Shani T, Ghanem N, Itzkovich C, Slomovich S, et al. Ex utero mouse embryogenesis from pre-gastrulation to late organogenesis. Nature [Internet]. 2021 Mar 17 [cited 2024 Feb 5];593(7857):119–24. Available from: https://www.nature.com/articles/s41586-021-03416-3. 33731940 10.1038/s41586-021-03416-3

[230] Zhang L, Getz SA, Bordey A. Dual in Utero Electroporation in Mice to Manipulate Two Specific Neuronal Populations in the Developing Cortex. Front Bioeng Biotechnol. 2022 Jan 12;9:814638. doi: 10.3389/fbioe.2021.814638 35096799 PMC8790278

[231] Huang Q, Cohen MA, Alsina FC, Devlin G, Garrett A, McKey J, et al. Intravital imaging of mouse embryos. Science (1979) [Internet]. 2020 Apr 10 [cited 2024 Feb 5];368(6487):181–6. doi: 10.1126/science.aba0210 32273467 PMC7646360

